# Functional stratification of cancer drugs through integrated network similarity

**DOI:** 10.1038/s41540-022-00219-8

**Published:** 2022-04-19

**Authors:** Seyma Unsal-Beyge, Nurcan Tuncbag

**Affiliations:** 1 Graduate School of Informatics, Middle East Technical University, Ankara, 06800 Turkey; 2 Department of Chemical and Biological Engineering, College of Engineering, Koc University, Istanbul, 34450 Turkey; 3 School of Medicine, Koc University, Istanbul, 34450 Turkey

**Keywords:** Computational biology and bioinformatics, Systems biology

## Abstract

Drugs not only perturb their immediate protein targets but also modulate multiple signaling pathways. In this study, we explored networks modulated by several drugs across multiple cancer cell lines by integrating their targets with transcriptomic and phosphoproteomic data. As a result, we obtained 236 reconstructed networks covering five cell lines and 70 drugs. A rigorous topological and pathway analysis showed that chemically and functionally different drugs may modulate overlapping networks. Additionally, we revealed a set of tumor-specific hidden pathways with the help of drug network models that are not detectable from the initial data. The difference in the target selectivity of the drugs leads to disjoint networks despite sharing a similar mechanism of action, e.g., HDAC inhibitors. We also used the reconstructed network models to study potential drug combinations based on the topological separation and found literature evidence for a set of drug pairs. Overall, network-level exploration of drug-modulated pathways and their deep comparison may potentially help optimize treatment strategies and suggest new drug combinations.

## Introduction

Transformation of normal cells to tumor cells is a multi-stage process where multiple signaling pathways and biomolecular connections alter^1–7^. Response to drug treatment is highly dependent on cellular and physiological factors in cancer, and many drugs have multiple targets^8^. Molecular heterogeneity across tumor types may result in different signaling alterations in response to the same drug^9–11^. Moreover, drugs simultaneously perturb multiple pathways besides their immediate targets. Therefore, network-based approaches may unveil many unknowns about drug response across various cancer types^12–14^. Integrative multi-omic approaches can provide a realistic view of network-level alterations toward developing better treatment strategies against the complexity and heterogeneity of cancer^15–19^.

Network-based strategies were previously used to investigate protein–drug and protein–protein interactions. Topological separation of disease modules within the human interactome was studied to elucidate disease–disease interactions^12^. In a similar line, network proximity of drug targets in the human interactome was shown to predict drug–disease interactions for drug repurposing^13^. Cheng et al. calculated the closest distances between drug targets and disease genes and used patient-specific data to investigate the effects of drugs on different diseases^14^. Ritz et al. developed PathLinker to reconstruct signaling pathways, which finds multiple shortest paths from receptors to transcriptional regulators in a reference protein interactome^20^. Wu et al. used BioNetGen software^21^ to model a detailed rule-based biochemical network of VEGF-mediated eNOS signaling pathway and interpreted the effects of an angiogenic inhibitor (thrombospondin-1, TSP1)^22^. Halasz et al. studied signal transduction networks in colorectal cancer by integrated network reconstruction using a Bayesian mechanistic modeling algorithm^23^. Naldi et al. combined weighted shortest paths and random walk methods^24^, and Buffard et al. used this method to identify significant pathways by integrating phosphoproteomic data from cancer cells^25^. Scoring topological proximity between disease proteins in a reference interactome was previously used to prioritize disease genes, infer new drug targets, identify drug efficacy, and predict phenotypic outcomes^26–31^. One challenge in network-based approaches is the incompleteness of human interactome. It has many false negatives^32^ and is biased to well-studied proteins. Previous works have combined link prediction approaches with network-based studies to overcome this challenge^33–36^.

Many cancer drugs eventually lead to resistance and cause adverse effects when applied continuously and with high doses^37,38^. Therefore, combinatorial drug treatment approaches are rigorously studied to eliminate or reduce resistance, recurrence, and possible side effects^39–44^. Effective drug combinations can be predicted with the help of network topology-based analysis of drug–disease interactions and inference of affected pathways^45^.

These approaches have the overarching aim of better understanding molecular alterations in diseases, how drugs act within the cell, and finding the best treatment options. In this study, we further elaborate on perturbed networks to mechanistically understand the similarities and differences of drugs. Conceptually we (i) grouped cancer drugs at the network level beyond their immediate targets, (ii) evaluated the alterations of drug modulation in different cell lines, and (iii) suggested potential drug combinations based on topological separation of the networks. For this purpose, we used an integrative approach based on reverse engineering principles that combine a link prediction strategy to modify the underlying interactome and the solution of the prize-collecting Steiner forest problem to reconstruct drug and cell line-specific networks. All-pair comparison of the reconstructed networks shows that chemically and functionally different drugs may modulate shared pathways. We next considered these reconstructed networks for coherence with the available drug response data and possible drug combination prediction. Thus, these network models are rich resources for examining different aspects of drug actions at the pathway level in different cancer types.

## Results

### Overview of the method

**Fig. 1:**
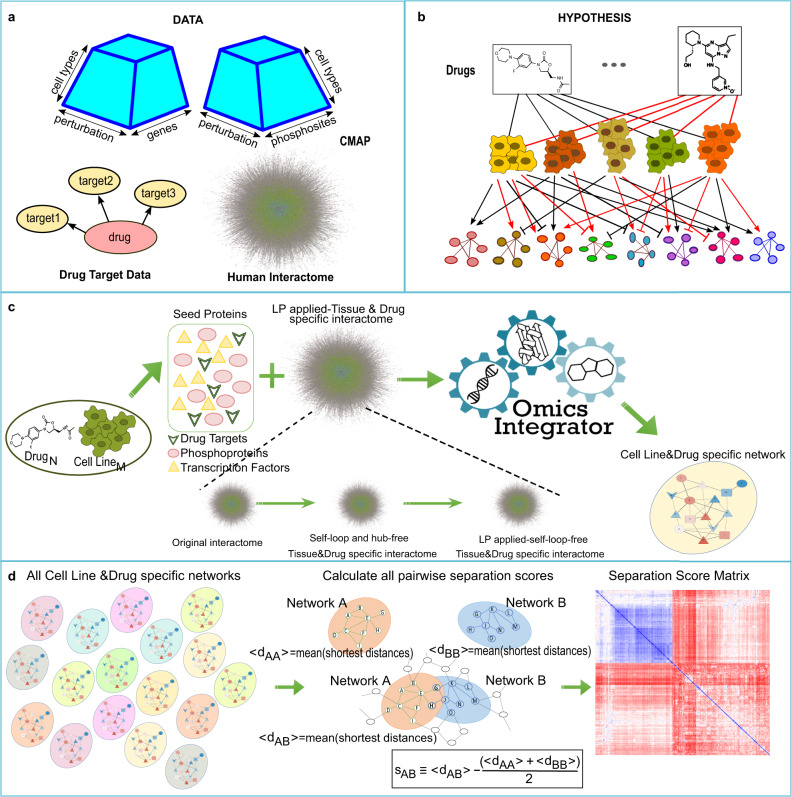
Overview of the study. **a** Initial data includes transcriptional and phosphoproteomic data of small molecule-perturbed five cancer cell lines, known drug-target proteins, and human protein–protein interaction network. **b** Each cell line-drug pair’s dataset is processed to reconstruct its corresponding subnetwork. Drugs modulate various pathways in different cancer cell lines, and induced cellular mechanisms of perturbed cancer cells can be revealed by omics data integration. **c** Omics Integrator software is used to discover the hidden connections underlying the effects of drugs, which needs two kinds of inputs: (i) seed proteins and (ii) reference interactome. Seed proteins are collected from the omics data in part (a). Reference interactome is processed with several approaches to decrease the impact of false positive and false negative interactions. Self-loops and lowly expressed genes are filtered. Afterwards, link prediction (LP) strategy is applied which is followed by cellular localization filters. Omics Integrator software produces optimal subnetworks for each cell line and drug-specific condition. **d** Finally, all subnetworks are collected, and pairwise separation scores are calculated to later use for comparison studies.

We used transcriptomic and phosphoproteomic data of five cancer cell lines treated with 89 drugs and the associated control treatment (Connectivity Map Project - CMap) to understand the drugs’ signaling level differences and commonalities systematically. We obtained the upstream regulators - the set of transcription factors—of the significantly expressed genes for each cell line-drug pair from transcriptomic data. Additionally, we retrieved the targets of each drug from CMAP Drug Repurposing tool^46^, which combines the data from DrugBank, PubChem, and other drug-related databases. Finally, we merged the set of transcription factors, phosphoprotein hits, and drug targets to obtain the list of seed proteins of each cell line-drug pair for the network modeling. In Fig. 1, we conceptually illustrate our integrative approach.

Although we started with 89 drugs and five cell lines, the number of proteins in the seed list is very low for some drugs, making the network modeling not feasible. We have also tested less stringent thresholds to enlarge the size of the seed list. However, we still could not overcome this issue for some drugs (i.e., decitabine, ginkgetin in A549 and YAPC, vemurafenib, and tacrolimus in MCF7 and PC3 cells). Therefore, we continued with 70 drug treatments on five cell lines. We calculated Tanimoto similarities and MACCS key distances of these 70 drugs (Supplementary Fig. 1 and Supplementary Note 1) to label their chemical similarities and their mechanism of action (Supplementary Fig. 2) to use as a reference in our analysis.

To discover the hidden mechanisms underlying the effects of drugs, we integrated the seed proteins list (drug targets, transcription factors, and phosphoproteins) with the human interactome using Omics Integrator^47^ software. Edge weights in the human interactome (iRefWeb v13.0) represent confidence scores of interactions and are calculated by the MI-Score function. We additionally refined the human interactome with several approaches to decrease the impact of false positive and false negative interactions. Additionally, the link prediction followed by cellular localization filters enriched the interactome (see Methods). Omics Integrator solves the prize-collecting Steiner Forest (PCSF) problem to reconstruct optimal subnetworks by integrating the list of seed proteins (terminal set) and a reference interactome. The performance of the network modeling approaches is highly dependent on the interactome, parameter tuning, and inclusion of known biological insights. Omics Integrator has a better performance compared to all pairs shortest paths, page rank, and heat diffusion approaches when rigorous parameter tuning is applied and multiple suboptimal solutions are merged. This modification increases the precision and recall of PCSF^48^. Additionally, PCSF can reconstruct a network from a single list of seed nodes whereas other powerful approaches such as PathLinker^20^ and ResponseNet^49^ require two sets of inputs. Because of its good performance among other methods requiring only a single seed list, we selected Omics Integrator as the main tool for network reconstruction. In this study, we merged the outputs of multiple parameter sets and refined the underlying interactome to reconstruct better networks. The terminal set size varies between 6 and 214 across the cell line-drug pairs, and the representation of phosphoproteins is relatively limited (max of 20 hits). Proteins have weights reflecting their importance in the terminal set based on their type. Transcription factors were weighted based on the mean of their target gene expressions. Phosphoproteins were weighted based on the absolute fold change compared to the control. Drug targets have a uniform weight inferred from the overall weight distribution of all proteins.

In total, we constructed 236 subnetworks from the combination of 70 drugs and five cell lines. However, not all cell lines have the same number of subnetworks. Out of 236 networks, cell line A375 has 70, A549 has 46, MCF7 has 43, PC3 has 59, and YAPC has 18 drug-specific subnetworks (Supplementary Fig. 3a). Topological characteristics of the drug networks can give clues about the connectivity of the modulated proteins. We summarized the topological properties of networks for each cell line in Supplementary Fig. 4. We found that the final network size is dependent on the number of altered proteins/genes from multi-omic data rather than the centrality of the drug targets in the interactome (summarized in Supplementary Note 2 and Supplementary Fig. 5).

We initially quantified the node level overlap between network pairs where extensive overlap means similar network neighborhood. 98.4% of network pairs share at least one protein. Therefore, a comparison solely based on the overlapping nodes and node frequencies does not allow us to understand the comprehensive modulation of drugs (Supplementary Fig. 3 and Supplementary Note 2).

The reconstructed networks preserve more detailed information about the drug effects beyond the common proteins and their association with cancer pathways. If two drug networks highly overlap, these drugs’ action and phenotypic outcomes may be potentially similar. We applied the network-based separation approach, developed by Menche et al.^50^, to reveal disease–disease relations on each drug network pair within and across cell lines to explore the network-level separation. The separation score represents how close two networks are. This topology-based comparison calculates the average shortest distances between the nodes in each network in the reference interactome (see Methods). The topological overlap of two drug networks in a cell line reflects their similarities at the pathway level. We found several overlapping subnetworks for drug pairs that do not have common target proteins or similar chemical structures, such as BIX-01338, entinostat, etoposide. The similarity between the networks modulated by these drugs was found statistically significant based on a hypergeometric test (Supplementary Data 1).

We next compared the application of the separation score method on reconstructed networks against the application on only seed proteins. The distribution of separation scores based on only seed proteins mostly in the negative range. The difference between two applications arises from the intermediate (Steiner) nodes added *via* the network reconstruction. These intermediate proteins can potentially reveal the off-target effects of drugs (Supplementary Fig. 6 and Supplementary Note 3).

Moreover, we investigated the contribution of link prediction results in reconstructed networks. We observed that predicted edges constitute very low percentages of total edge count in the networks, and they do not cause any spurious proteins to be included in the networks. On the other hand, predicted edges can be considered as the noise introduced to the reference interactome. Their small contribution to the reconstructed networks shows the robustness to this noise (Supplementary Note 4, Supplementary Table 1, and Supplementary Data 2).

### Chemically and functionally different drugs may modulate overlapping networks

**Fig. 2:**
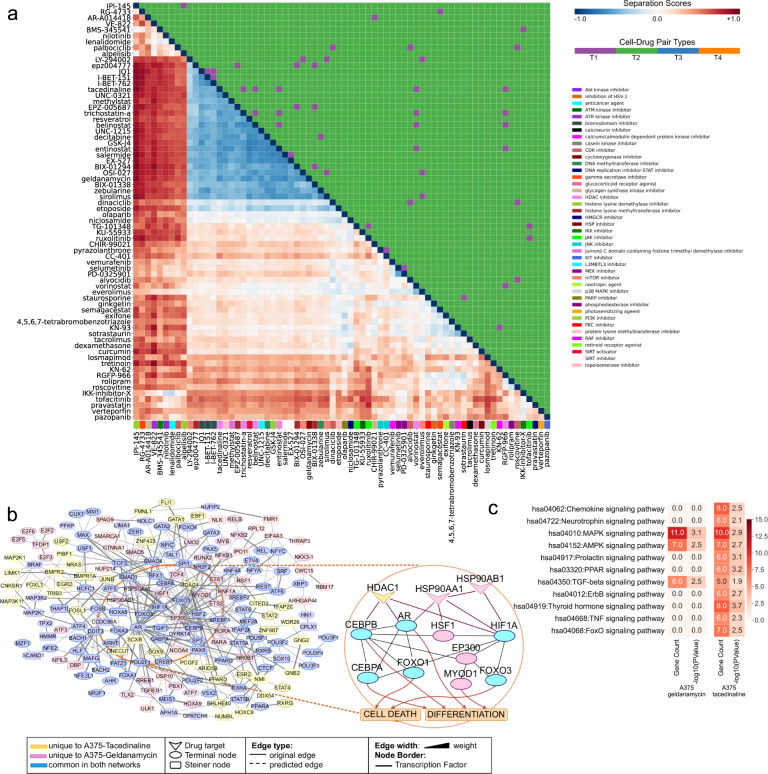
Cell line-based network separation score matrix and network comparison case study. **a** Heatmap showing network-based separation scores(s_AB_) among 70 drugs on A375 cell line. Drugs are hierarchically clustered based on the pairwise separation scores, and the lower diagonal is colored on the scale of s_AB_. The upper triangle of the heatmap highlights the four distinct classes defined based on the cell line and MoA types (T1: same cell types and MoAs; T2: same cell types but different MoAs; T3: different cell types but the same MoAs; T4: different cell types and MoAs). Colors on the x-axis refer to the MoA of each column mapped on the right of the heatmap. **b** Merged network maps of Tacedinaline and Geldanamycin on A375. Blue nodes represent proteins common in both networks. Yellow nodes are the proteins unique to Tacedinaline, and pink nodes are the proteins unique to Geldanamycin. Drug-target proteins, nodes coming from seed proteins, and intermediate (Steiner) nodes are shown as V-shaped, ellipse-shaped, and round rectangular-shaped, respectively. Edges present in iRefWeb human interactome are solid lines, and LP-predicted edges are dashed lines. Line width reflects edge weight. Nodes with borders are transcription factors. Sub-panel: downstream transcription factors of HDAC1 and HSP90s shared within cancer phenotypes (found using CancerGeneNet). **c** Signaling pathways enriched in Geldanamycin and Tacedinaline networks on A375.

Transforming the networks into a matrix of separation scores (Supplementary Data 3) and clustering represents the overlaps of the networks as drug modules. We observed that the network overlap of some drugs is very high in specific cell lines such as A375, MCF7, and PC3, although their chemical similarity is limited. All-pair separation scores of drug modules in A375 (skin melanoma) cell line are illustrated in Fig. 2a with corresponding MoA similarities (T1 = same MoA, T2 = different MoA). We noticed that the chemically and functionally different drugs modulate similar networks. Separation score of network pairs in other cell lines can be found in Supplementary Fig. 7. For example, we observed that tacedinaline and geldanamycin have overlapping networks in the A375 cell line, suggesting similar signaling output despite their different targets or mechanism of action (separation score = −0.61, Fig. 2b).

Tacedinaline is a selective HDAC1 inhibitor, while geldanamycin is a heat-shock protein (HSP) inhibitor targeting HSP90AB1 and HSP90AA1 proteins. Effects of HDAC inhibitor and HSP90 inhibitor drugs on HDACs and HSPs and the potential interplay between HDACs (especially HDAC6) and HSP90 has been highlighted in several studies^51–57^. In our analysis, two drugs share several downstream transcription factors (i.e., HIF1A, HSF1, CEBPA, CEBPB, AR, and FOXO1/3) in the reconstructed networks. We then linked these drug targets to cancer phenotypes using CancerGeneNet^58^ which calculates the shortest paths between genes and phenotypes. We found that the downstream transcription factors of HDAC1 and HSP90s are shared on the path toward cell death and differentiation phenotypes (Fig. 2b sub-panel). The intersection of tacedinaline and geldanamycin networks in A375 is enriched in several pathways such as MAPK, AMPK, and TGF-beta signaling pathways. Moreover, transcriptomic data of two drugs are highly correlated. Despite the high overlap, they have some disjoint pathways enriched in each specific network. Tacedinaline affects chemokine, neurotrophin, ErbB, TNF, FoxO, PPAR, prolactin, and thyroid hormone signaling pathways that are not present in the geldanamycin network (Fig. 2c).

To systematically evaluate the performance of this method, we classified the drug pairs having the same MoA and similar resulting networks as true positives (TP) and drug pairs having different MoA and overlapping networks as false positives (FP). We defined drug pairs having the same MoA and separated networks as false negatives (FN) and drug pairs having different MoA and separated networks as true negatives (TN). We plotted the ROC curve and Precision-Recall curve using different threshold values (Supplementary Fig. 8 and Supplementary Data 4). Because the data is imbalanced (200 positive cases—pairs with same MoA, 6017 negative cases—pairs with different MoA), the best performance is found based on Matthews Correlation Coefficient (MCC). The best performance is achieved when the separation score threshold is selected as −0.45. (precision = 0.138, recall = 0.400, accuracy = 0.900, sensitivity = 0.400, specificity = 0.917, TPR = 0.400, FPR = 0.083, F1-score = 0.205, MCC = 0.192). Same MoA and overlapping networks are expected, but the interesting cases are the drug pairs with different MoA and overlapping networks. We, therefore, further studied the literature to find evidence about mechanistic similarities among FP drug pairs. This evidence strengthens our claim that even drugs with different MoA can perturb similar pathways (Supplementary Table 2). Despite being an FP based on the ground-truth, the synergistic behavior of decitabine and entinostat was previously shown in the activation of FoxO1^59^.

There are network pairs following the ground-truth, i.e., drugs having the same MoA and overlapping networks. One example of drugs with the same MoA is sirolimus and OSI-027, which are selective mTOR inhibitors. Phase I clinical trials of OSI-027 were completed in 2013 for the investigation of patients with advanced solid tumors or lymphoma. It has activity on pancreatic ductal adenocarcinoma as an inhibitor of cell proliferation^60^. The networks of sirolimus and OSI-027 in A375 cells highly overlap (ss = −0.562). They have many common proteins and shared signaling pathways such as MAPK signaling, PI3K-Akt signaling, and cGMP-PKG signaling pathways. However, the OSI-027 network has specifically an enrichment of Hippo signaling, Jak-STAT signaling pathways, while sirolimus differs from OSI-027 with the enrichment of Wnt signaling, ErbB signaling pathways, and some immunity-related pathways such as Toll-like receptor signaling pathway (Supplementary Fig. 9A, B).

Selumetinib and PD-0325901(Mirdametinib) are MEK inhibitors targeting MAP2K1. We showed a consistent similarity between two drugs for the same cancer types. The separation scores between two drugs reach up to −0.64 in YAPC (pancreatic cancer) (Supplementary Fig. 9C, D). However, these drugs may perturb separated networks in different cancer types. For example, the separation score between PD-0325901 in the PC3 cell line and selumetinib in the YAPC cell line is 0.48 that implies dissimilar networks. While the affected pathways are FoxO, VEGF, ErbB cAMP, Rap1, Ras, and GnRH signaling pathways in PD-0325901 treated PC3 network, Jak-STAT, TGF-beta, PI3K-Akt, TNF, and Wnt signaling pathways are enriched in selumetinib treated YAPC network (Supplementary Fig. 9E, F).

### Target selectivity of the drug pairs determines the network separation, despite having the same MoA

**Fig. 3:**
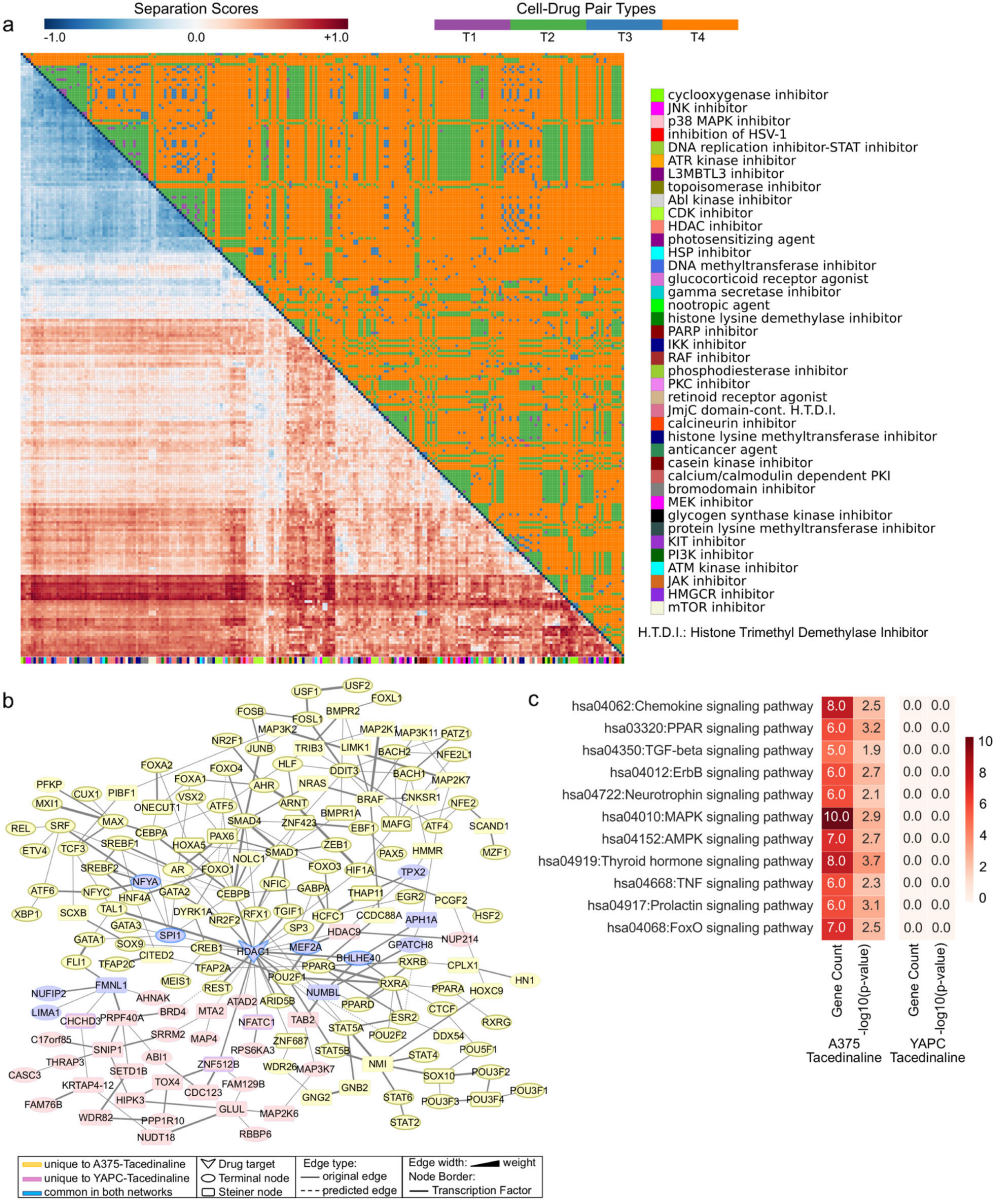
Network separation score matrix and a network comparison case study for Tacedinaline on two different cell lines. **a** Heatmap of network-based separation scores(s_AB_) among 236 cell line-drug pairs. Drugs are hierarchically clustered based on the separation scores and color keys are shown by s_AB_. The upper triangle of the heatmap highlights the four distinct classes defined based on the cell line and MoA types (T1: same cell types and MoAs; T2: same cell types but different MoAs; T3: different cell types but the same MoAs; T4: different cell types and MoAs). Colors on the x-axis refer to the MoA of each column mapped on the right of the heatmap. **b** Merged network maps of Tacedinaline on A375 and YAPC. Blue nodes represent proteins common in both networks. Yellow nodes are the proteins unique to A375, and pink nodes are the proteins unique to YAPC. Drug-target proteins, nodes coming from seed proteins, and Steiner nodes are shown as V-shaped, ellipse-shaped, and round rectangular-shaped, respectively. Edges present in iRefWeb human interactome are solid lines, and LP-predicted edges are dashed lines. Line width reflects edge weight. Nodes with borders are transcription factors. **c** Signaling pathways enriched in Tacedinaline networks on A375 and YAPC.

The analysis of drug modulation in a cell line-specific manner helped determine a subset of drugs acting similarly in A375, MCF7, and PC3 (Supplementary Data 5). Afterward, we measured the separation of the drugs across different cell lines to discover if the same subset of drugs similarly acts in various tumor types. We compared the separation scores of drug pairs from 236 networks (Fig. 3a) by dividing them into the same cell line and the different cell line groups. Next, we checked if two similar drugs in the same cell line act similarly in different cancer types. The results show that the network level overlap between drug pairs in the same cell line is preserved across different cell lines, but their separation is relatively higher. For example, resveratrol and geldanamycin were highly overlapping in the A375, MCF7, and PC3 cell lines (in A375, separation score = −0.60; in MCF7, separation score = −0.47; in PC3, separation score = −0.54), but their overlap is lower across cell lines such that separation score between A375-resveratrol and MCF7-geldanamycin is −0.40 and separation score between A375-resveratrol and PC3-geldanamycin is −0.43. Another aspect of this difference is the target selectivity of the drugs. Two examples of this phenomenon are the HDAC inhibitors and JAK inhibitors.

RGFP-966 is a slow-on/slow-off, competitive and selective HDAC3 inhibitor. Tacedinaline selectively targets HDAC1. Other HDAC inhibitors are Belinostat, Entinostat, Trichostatin-a, and Vorinostat. Network separation scores of these HDAC inhibitors across the cell lines vary based on the target selectivity. We observed both overlapping and non-overlapping networks depending on the cell types. Belinostat, Trichostatin-a, and Vorinostat networks usually have partially overlapping regions in all cell types, as these drugs are in the same structural group, and expected to behave similarly. Tacedinaline treatment activates similar mechanisms on A375, MCF7, and PC3 cells and separation scores are negative. However, network of Tacedinaline on YAPC cells is relatively distant to an other cell networks. Separation score of Tacedinaline networks on A375 and YAPC cells is 0.38. YAPC-Tacedinaline network is a small network. It is not significantly enriched for any pathways other than Osteoclast differentiation which is recently shown to be induced in pancreatic cancer-derived exosomes^61^. In contrast, the A375-Tacedinaline network is enriched in several critical signaling pathways such as MAPK, ErbB, FoxO, TGF-beta, and TNF signaling pathways (Fig. 3b, c). However, RGFP-966 treatment on A375, MCF7, and PC3 results in separated networks that are distant to each other and to the networks of other HDAC inhibitors. RGFP-966 networks have very low overlapping regions across different cell lines (MCF7-PC3:0.42, A375-PC3:0.28, and A375-MCF7:0.45). Selectivity of RGFP-966 on HDAC3 among all HDAC proteins may result in the induction of various pathways in different cell types. When RGFP-966 is compared to Belinostat, Entinostat, Trichostatin-a, and Vorinostat, the most distant networks have a separation score around 0.4, and these networks generally belong to A375 and PC3 cells. For example, the PC3-RGFP-966 network is separated from A375-Trichostatin-a with a score of 0.46. Two networks are not commonly enriched in any pathway, while PC3-RGFP-966 is only enriched in the cell cycle, but A375-Trichostatin-a is enriched in estrogen, neurotrophin, and TNF signaling pathways (Supplementary Fig. 10A, B). As Tacedinaline is the other selective HDAC inhibitor, RGFP-966 and Tacedinaline treatments cause more distant PPI networks. RGFP-966 in PC3 and Tacedinaline in A375 networks are the most distant pair with a separation score of 0.62. They only have three proteins in common, and there are no commonly affected pathways (Supplementary Fig. 10C, D).

Among three JAK inhibitors, ruxolitinib, tofacitinib, and TG-101348 (fedratinib), only TG-101348 is known for its selectivity to JAK2 protein. The rest targets with varying affinities JAK1, JAK2, JAK3, and TYK2 proteins^62–67^. According to our results, same drug may modulate different subnetworks in different cell lines, but still by keeping a marginal overlap. To facitinib and ruxolitinib networks have the most distant network pairs with separation scores of 0.68 in A375 and 0.46 in PC3, respectively. In A375, they only share the target proteins, JAK1 and JAK2. The tofacitinib network differs from ruxolitinib with the enrichment of Jak-STAT, ErbB, and PI3K-Akt signaling pathways. In PC3, they share two more proteins other than JAK1/2 while the ruxolitinib network is enriched in the AMPK signaling pathway, and the tofacitinib network is only enriched in the Jak-STAT signaling pathway. A relatively low separation score (−0.147) is obtained from networks of TG-101348- and tofacitinib-treated A549 cells. Two networks share 23 proteins besides the target proteins, and commonly induced pathways include Jak-STAT and Notch signaling pathways. However, TG-101348 and tofacitinib networks have a separation score of 0.4 in A375, and the Jak-STAT signaling pathway is commonly enriched while the A375-tofacitinib network differs from TG-101348 with ErbB, mTOR, PI3K-Akt signaling pathways (Supplementary Fig. 11).

### Topologically separated drug networks may guide the use of drug combinations

**Fig. 4:**
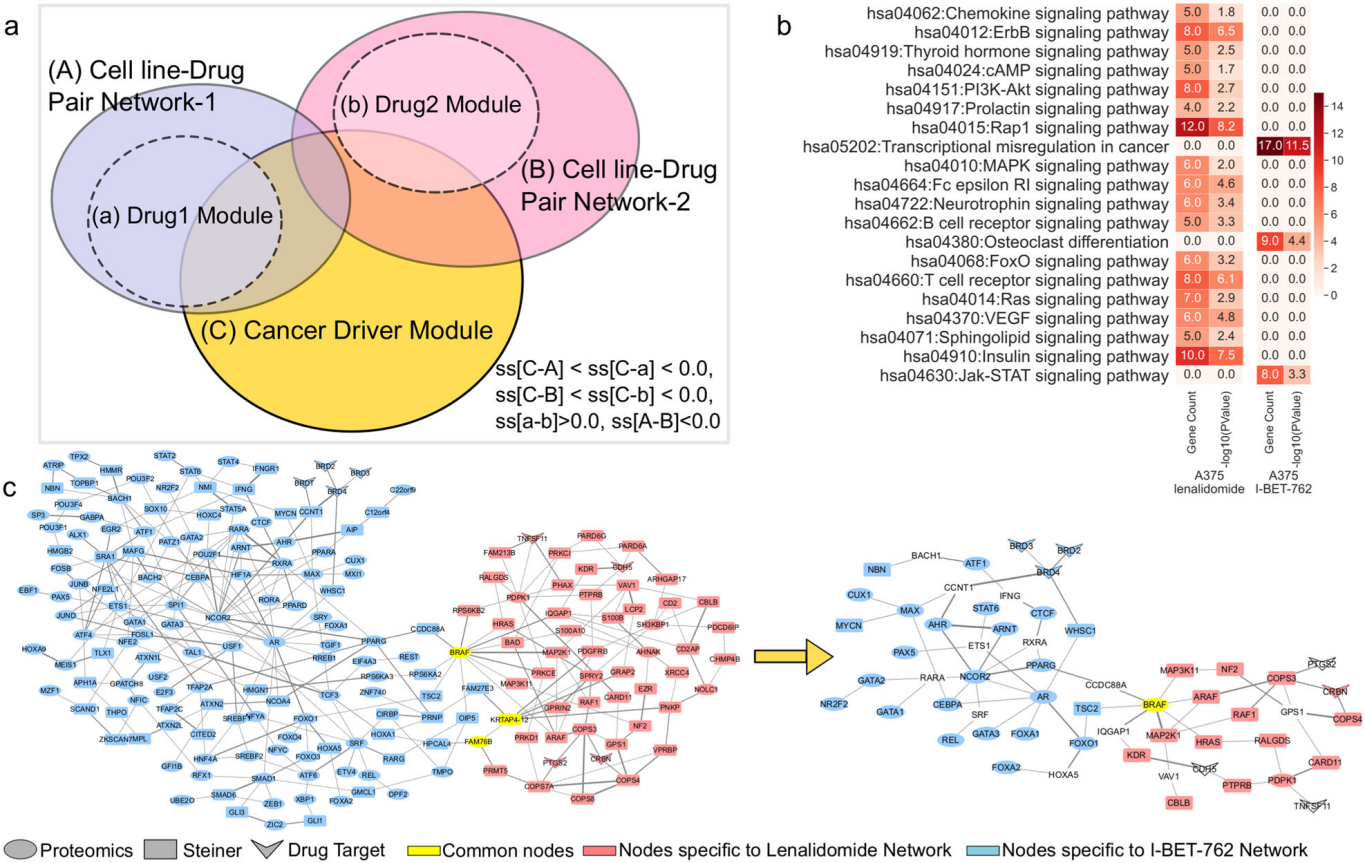
A case study for combinatorial cancer drug pairs. **a** The overlaps of drug and disease modules together with cell line-drug pair networks. **b** Signaling pathway enrichments of Lenalidomide and I-BET-762 networks on A375. **c** Merged network maps of Lenalidomide and I-BET-762 treated A375 cell lines and the subnetwork that includes cancer driver genes. Yellow nodes represent commonly found proteins in two cell line-drug pair networks, blue nodes represent proteins unique to the A375-I-BET-762 network, and pink nodes represent proteins unique to the A375-Lenalidomide network.

Modulating orthogonal sets of proteins or pathways simultaneously is an effective strategy for finding drug combinations. Modulated pathways are usually topologically close enough to perturb the disease module commonly. This can be described as targeting a disease through multiple signaling pathways^11,68^. Therefore, networks play an essential role in identifying drug combinations. Cheng et al. showed the efficacy of drug combinations using drug targets and disease proteins by calculating their separation mapped on the interactome^45^. Given two drugs, if each drug module overlaps with a disease module and these two drug modules are topologically separated, this is defined as complementary exposure^45^. They demonstrated six classes of drug combination-disease interactions from FDA-approved or experimentally validated pairs and found one class correlates with therapeutic effects (complementary exposure). In our study, we found the intersection between our network models and the drug combinations provided in Cheng et al. Our reconstructed drug networks include many hidden proteins and modulated omic hits besides the drug targets. Therefore, the coverage of the networks is higher than the drug modules described in previous studies. In our analysis, we considered the disease module as the set of cancer driver genes (obtained from Cancer Genome Interpreter^69^). We defined a rule where if two drug networks have at least one cancer driver protein in common and each has a topologically disjoint region, then the combination of these drugs can be effective. (Fig. 4a). We only have four drug pairs intersecting with Cheng et al., but when we consider them per cell type, we could analyze seven drug pairs in total.

The separation scores of seven cases vary between −0.09 and 0.675. Network pairs in this analysis share a limited number of nodes (min three, max 16 proteins). KEGG pathway enrichment of these drug networks shows that each drug modulates a different region of the interactome so that different signaling pathways are perturbed. Therefore, we suggest that their combination may be effective (Supplementary Figs. 12, 13).

Considering these seven cases and the concept of complementary exposure as a guideline, we rated other drug pairs in our dataset with similar criteria. If two networks are separated (ss >0.0) and share less than two common enriched pathways and the networks are not in small size (number of nodes >40), and at least one is a large network (number of nodes >100), then these drug pairs have potential to be used in combination. Given these criteria, we found 1441 potential drug combinations out of 6217 possible cell line–drug network pairs. Some of these drug pairs were previously supported in the literature for their use in combination therapy. The top-ranking drug pair was I-BET-762 and lenalidomide in A375 cell line. Two networks are separated with a score of 0.693, and they do not share any common signaling pathways. Lenalidomide network is enriched in several essential pathways such as PI3K-Akt, ErbB, MAPK, Ras, Rap1, VEGF, and FoxO signaling pathways, while the I-BET-762 network is enriched in Jak-STAT signaling and transcriptional misregulation in cancer pathways (Fig. 4b). Three proteins are shared between these networks. Among them, “BRAF” is a cancer driver protein. These networks also contain different disease-related proteins (Fig. 4c). The combination of lenalidomide with CPI-203, a primary amide analog of (+)-JQ1 and having the same mechanism of action as I-BET-762, is shown to synergistically induce cell death in bortezomib-resistant mantle cell lymphoma^70^. Similar activities of BET inhibitors and lenalidomide are reported in different studies of multiple myeloma^71,72^.

Other examples within the top-ranking 100 predicted drug pairs are geldanamycin and tofacitinib (A375, ss = 0.656), trichostatin-a and tofacitinib (A375, ss = 0.562), and resveratrol and ginkgetin (PC3, ss = 0.53). HSP90 and JAK2 inhibition was shown to synergistically overcome resistance to JAK2-TKI in human myeloproliferative neoplasm cells^73^. Tofacitinib targets JAK proteins nonspecifically; however, selective JAK2 inhibitor TG-101348 was also predicted in combination with tofacitinib. Moreover, HDAC and JAK dual inhibition were previously studied to improve treatment strategies^74–77^. Resveratrol and ginkgetin combination was also shown to suppress VEGF-induced angiogenesis in colorectal cancer synergistically^78^.

### Topological separation between drug networks across cell lines gives clues about their sensitivity levels

Next, we associated the network models with the drug sensitivity of the cell lines. For this purpose, we collected drug sensitivity scores of cell lines deposited in the Genomics of Drug Sensitivity in Cancer (GDSC) Database^79^. The intersection of GDSC and CMap datasets results in five drugs that at least one cell line is significantly resistant or sensitive to. A375 is sensitive to CHIR-99021 and PD-0325901. CHIR-99021 has reconstructed networks in three cell lines (A375, MCF7, and PC3). PD-0325901 has reconstructed networks in four cell lines (A375, A549, PC3, and YAPC). Trichostatin-a has networks in two cell lines (MCF7 and A375), and MCF7 is significantly sensitive to Trichostatin-a. Thus, we can compare these networks to better understand the changes in sensitivity of cell lines to these drugs. PC3 is resistant to Staurosporine and YAPC is resistant to Dinaciclib. Since there may be several processes underlying the resistance of cells and we can not directly infer these mechanisms from our reconstructed networks, we focused only on the drugs that cell lines are sensitive to (CHIR-99021, PD-0325901, and Trichostatin-a).

**Fig. 5:**
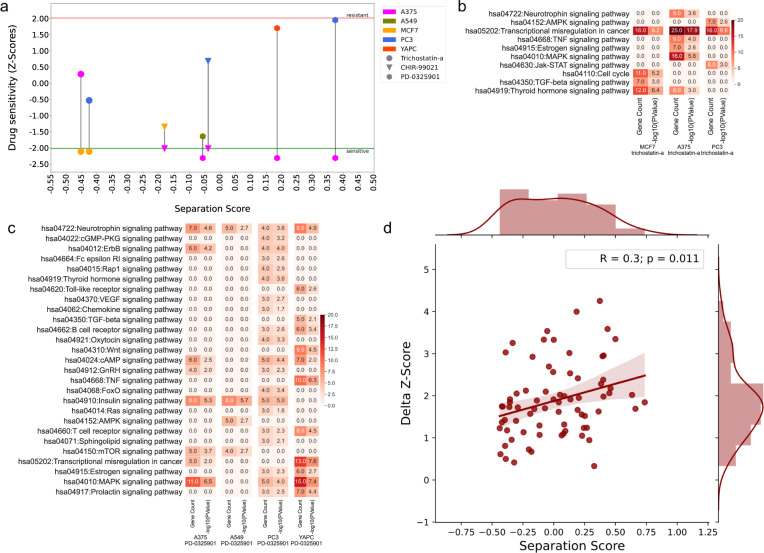
Analysis of drugs that cell lines are sensitive to. **a** Separation scores for network pairs of each drug in sensitive cell lines and non-sensitive cell lines versus drug sensitivity plot. Drugs are shown in different shapes and cell lines are shown in different colors. **b**, **c** Signaling pathways enriched in drug networks per cell type: Trichostatin-a and PD-0325901, respectively. **d** Regression plot of pairwise separation scores and drug sensitivity score differences for conditions in which one drug of two cell line-drug pair networks has a negative *z*-score on its corresponding cell line and the other drug has a positive *z*-score on its corresponding cell line and in which the separation score is higher than −0.45.

If a drug has dissimilar network models across different cell lines, sensitivity to that drug may also vary (Fig. 5a). Since Trichostatin-a(TSA) has highly overlapping networks across three cell lines, we could not observe this pattern for sensitive MCF7 across A375 and PC3. MCF7-TSA network differs from others with active TGF-beta signaling and cell cycle pathways, while A375 is differently enriched in MAPK signaling, neurotrophin signaling, estrogen signaling, TNF signaling pathways, and PC3 differs with Jak-STAT signaling and AMPK signaling pathways. TSA targets class I and class II HDACs. Treatment with HDAC inhibitors is reported to restore TGF-beta signaling in breast cancer^80^, so it is expected to observe that the TGF-beta signaling pathway is enriched in MCF7 cells (Fig. 5b). The higher difference between *z*-scores implies the more separated networks in CHIR-99021 and PD-0325901 modulation. Therefore, analyzing the networks of these drugs and exploring enriched pathways may give clues about the resistance to these drugs. PD-0325901, to which A375 cells are sensitive, is a selective MAP2K1 (MEK1) inhibitor directly related to cell proliferation. Given the MEK1 is central in RAS/RAF/MEK/ERK pathway, it is upstream of several cellular mechanisms for cell proliferation and cell survival. In networks of two cell lines whose *z*-scores are close to the resistance threshold, YAPC and PC3, we observed several modulated pathways as expected. While the neurotrophin signaling pathway is enriched in all cells, PC3 cells differ with several pathways such as cGMP-PKG signaling, sphingolipid signaling, Rap1, Ras signaling, VEGF signaling, Oxytocin signaling, FoxO signaling, and Fc epsilon RI signaling. Sphingolipid signaling is shown to be involved in the resistance of prostate cancer cell lines to antineoplastic drug treatment (*z*-score of PC3 is 1.95, very close to the resistance threshold)^81^ (Fig. 5c).

We also analyzed all possible relationships between *z*-score differences and separation scores. We could find *z*-scores of 30 drugs and calculated pairwise *z*-score differences of those with cell line-drug networks and their associated separation scores. After filtering for pairs with one negative *z*-score and one positive *z*-score, and separation scores higher than −0.45, we performed a regression analysis and observed a moderately positive correlation such that separation score increases with increasing delta *z*-scores (*R* = 0.29, *p* value = 0.011) (Fig. 5d).

## Discussion

Modulation of drug targets is not local when the complex interactions between molecules are considered within the cell. The impact can diffuse to distant parts or modulate several pathways simultaneously. Drugs perturb their specific network, which can be used to detect pathway-level similarities between drugs. We, therefore, reconstructed networks of cancer drugs by integrating transcriptomic, phosphoproteomic, and drug targets with protein–protein interactions using Omics Integrator. We modified the underlying reference interactome with gene expression data and predicted interactions to have a cell line-specific interactome. The resulting drug networks recover hidden intermediate proteins besides the initial seed proteins to explore their connectedness. We hypothesize that the topological overlap between the drug network pairs on the reference interactome can be a solid basis for identifying their pathway-level similarities. Our topological and pathway-level analysis of 236 reconstructed networks from five cell lines and 70 drugs demonstrated that chemically and functionally different drugs may modulate overlapping networks, which can not be revealed conventional comparisons based on drug properties. For example, tacedinaline and geldanamycin target different proteins, but their networks in A375 cell line share a module consisting of common transcription factors leading to cell death and differentiation phenotypes. Another example is drugs targeting same proteins and modulating separated networks i.e., sirolimus and OSI-027. Sirolimus network is enriched in Wnt, cAMP, ErbB, and FoxO signaling pathways, while OSI-027 network is differently enriched in Jak-STAT and Hippo signaling pathways in A375 cells. Besides exploring the different drugs in the same tumor type, we can also compare the network-level impact of a drug across multiple cell lines. Tacedinaline, one of the HDAC inhibitors, modulates MAPK, ErbB, FoxO, TGF-beta pathways in the A375 cell line in the reconstructed network while these pathways are absent in the network of YAPC cell line. Additionally, the target selectivity of the drugs is a strong signature of the separation of the networks. We, therefore, specifically investigated the networks of HDAC inhibitors and JAK inhibitors. Networks of drugs, such as vorinostat, belinostat, trichostatin-a, targeting the same HDAC proteins have overlapping regions in almost all cancer cells. On the other hand, the network of RGFP-966, which targets HDAC3 selectively, is topologically distant from other HDAC inhibitor networks in different cancer types.

As evidenced by Menche et al.^50^ that the overlap of the disease modules can quantify the molecular similarity of the diseases, we applied this quantification to interpret the drug similarities and eventually the effects of potential drug combinations. Treatment strategies combining drugs are used to decrease adverse effects and to prevent resistance to drugs^82–85^. Therefore, we compared our drug network models with experimentally validated drug combinations. Recently, the DREAM AstraZeneca-Sanger drug combination prediction challenge provided a large combinatorial cell line screening dataset. The challenge was open to methods to predict synergistic drug combinations, i.e., machine learning or network-based approaches^86–89^. In this study, we found that the experimentally synergistic drug pairs modulate topologically separated networks. Using their separation scores, common pathway enrichments, and network sizes, we rated cell line–drug network pairs and found 1441 drug pairs out of 6217 possible pairs. These predictions can be further used to experimentally study their synergy on specific conditions and provide guidance to finding alternatives to currently used drug pairs. In the future, we will integrate our network-level results with learning-based approaches to better understand the pharmacodynamics of drugs.

Another use of drug network models is to understand the drug resistance mechanisms. To reveal the differences between sensitive and resistant cells to a given drug, we compared their reconstructed networks. Cell lines' networks, that are perturbed by the same drug and have different response, have limited or no topological overlap. Therefore, pathway-level comparison and the topological analysis of drug networks may potentially guide in drug response predictions.

In summary, we leveraged networks to find similarities and disparities between drugs within and across multiple cancer cell lines. Further, these reconstructed networks are used for understanding drug response and combinations. We believe that network-based approaches are fundamental in interpreting the drug actions in tumors and integrating multi-omic data. This study was directly devoted to this task.

## Methods

### Data

The “omic” data is obtained from Connectivity Map (CMap), which contains 1.3 M L1000 data for nine different core cell types, treated with 27,927 perturbagens. Within CMap, the Touchstone V1.1 dataset contains 8388 perturbagens (well-annotated genetic and small-molecular perturbagens) for nine cell lines for three time points and three replicates; and P100 dataset contains proteomic data for six different core cell types, treated with 90 perturbagens^90,91^. In addition to transcriptional data (L1000), proteomic and phosphoproteomic data for a subset of these perturbagens have been recently released (P100, released on 4/13/18). P100 dataset was used in our study, which contains already filtered transcriptional and phosphoproteomic data for the same set.

The P100 dataset represents three different read-outs (phosphosignaling (P100), chromatin modifications (GCP), and transcriptional changes(L1000)) of the same 90 small-molecule perturbations in six cell lines. The duration of treatment was 3 h for P100, 24 h for GCP, and 6 h for L1000. These data were available at multiple levels of processing: level 1 is fluorescence intensity (for L1000) or mass spectrometry extracted ion chromatogram traces (for P100, GCP); level 2 is gene expression or proteomic values without normalization; level 3 is normalized; and level 4 is differential (i.e., each sample is compared to all other samples on a plate). We used normalized (level3) data of L1000 and P100 for five cancer cell lines in our studies.

L1000 assay measures transcription levels of 978 genes (referred to as Landmark genes) and 80 control transcripts directly. It then infers the expression levels of the remaining 11,350 genes via Ordinary Least Square (OLS) regression. From 11,350 inferred genes, 9196 genes are considered well inferred and called Best Inferred Genes (BING). We used landmark genes in the terminal set preparation procedure. Phosphoproteomic data generated by the P100 assay consists of 96 phosphosites and is a reduced representation of phosphoproteomics, targeting common signaling pathways.

The drug-target interactions are retrieved mainly from CLUE Drug Repurposing tool^46^, which curates information from several databases such as DrugBank & PubChem (https://clue.io/repurposing-app). The reconstructed networks have been oriented through these targets for each drug.

We also have the edge-weighted protein–protein interaction network for the network modeling part retrieved from iRefWeb, v13.0 reference human interactome, which has 15,404 nodes (proteins) and 175,820 weighted edges (protein interactions) without self-loops. The weights of edges represent how real interaction is based on the MI-Score function.

### Calculation of Tanimoto and MACCS key distance similarities

For 70 drugs listed with network reconstruction studies, SMILES signatures are collected from chemical databases such as PubChem and DrugBank. All pairwise Tanimoto and MACCS key similarities are calculated with the open-source RDkit Python module^92^. Hierarchical clustering on similarity matrices is performed with the Morpheus tool of BROAD institute with the parameters as the metric is “Euclidean distance” and linkage method is “average” (https://software.broadinstitute.org/morpheus/).

### Network modeling approach

For network reconstruction purposes, a solution to the prize-collecting Steiner tree problem was searched through Omics Integrator software^19^ to find an optimum tree. Nodes obtained from the experiments (terminal nodes) and nodes not detected in experiments and obtained by the algorithm (Steiner nodes) were determined by this process. Let G = (V, E, c, p) be an undirected graph, with the vertices/nodes V associated with non-negative prizes p(v), and with the edges E associated with non-negative costs c(e). The Prize-Collecting Steiner Tree problem (PCST) consists of finding a connected subgraph T = (V′, E′) of G, that minimizes the weight of T, which is the sum of its edge costs plus the sum of the penalties of the vertices of G outside of the solution.

Omics Integrator is a software package that applies the prize-collecting Steiner forest (PCSF) approach to construct the most biologically relevant protein–protein interaction network. This tool is efficient in the integration of different omics data using interactome data.

There are two distinct tools within the Omics Integrator package: Garnet and Forest. To integrate different experimental results derived from mRNA, proteins, metabolites, etc., measurements, Garnet and Forest complement each other and use omics data either gathered from experiments or derived from several databases. In this study, only the Forest tool was used.

Forest tool constructs the interaction network by using user-defined omic data that is the list of proteins/genes together with their importance(prizes), and by using interactome data together with their significance levels. Each protein/gene given as input to the Forest tool is defined as “terminal”. If necessary, Forest can add extra nodes from the interactome data called “Steiner” nodes. When constructing the network, the algorithm optimizes the score by calculating the sum of prizes of nodes not included plus the costs of edges included. The algorithm seeks to minimize the score to find the most optimum and biologically relevant protein–protein interaction network. There is always a possibility to include “hub” nodes, highly-connected nodes. Forest uses a generalized prize function to decide whether these “hub” nodes are, in fact, essential and should be included in the network or not. This generalized prize function assigns negative weights to nodes according to the number of connections they have in the interactome. The function is:

1 $$p{\prime}\left( v \right) = \beta \cdot p\left( v \right) - \mu \cdot degree\left( v \right)$$

where *β* and *µ* are scaling parameters and degree(v) is the number of connections of node v in the interactome. *β* is used to calibrate the effect of terminal nodes, and *µ* is used to calibrate the effect of hub nodes. *µ* is 0 in the default parameters where hub correction is disabled; if it is increased, the algorithm tries to exclude these hub nodes. *β* enables the algorithm to include terminal nodes, and increasing *β* facilitates more terminal nodes to be included in the final network.

Given a directed, partially directed, or undirected network G(V, E, c(e), p′ (v)), it is aimed to find a forest F(V_F_, E_F_) that minimizes the objective function:

2 $$f{\prime}\left( F \right) = \mathop {\sum}\nolimits_{v \notin V_F} {p{\prime}\left( v \right)} + \mathop {\sum}\nolimits_{e\smallint E_F} {c\left( e \right) + \omega \cdot \kappa }$$

where p′ (v) is defined in Eq.1, c(e) is the cost of each edge, κ is the number of trees in the forest, and *ω* is another scaling parameter which is a uniform edge cost of each node connected to dummy node.

The forest tool has six PCSF parameters; however, *ω*, *β*, and *D* are the ones that at least need to be defined by the user. *D* is the depth parameter which is the maximum path length from v_0_ to terminal nodes. The other three optional parameters are *µ*, *g* (reinforcement parameter, default is 1e-3), and *garnetBeta* (scales the Garnet output prizes relative to the provided protein prizes, default is 0.01).

Transcriptional and phosphoproteomic datasets (P100) were used to model protein–protein interaction networks. Gene expression data allows detecting transcription factors while phosphoproteomic data allows differentiating active and inactive proteins. By integrating these two data types and drug-target data, multilayer networks were constructed. For parameter tuning purposes, we run the algorithm for each combination of D = 10, *µ* = [0.00, 0.005, 0.01, 0.015, 0.02, 0.025, … , 1.0]*, β* = [2, 3, 4, 5, 10], and *ω* = [1, 2, 3]. After collecting all the networks, we chose the ones with the highest number of terminal nodes and the minimum number of “hub” proteins (degree >100) and then merged all of these networks to get the final cell line-drug network.

### Preparation of terminal sets as an input for network reconstruction method

All statistical analyses were performed with the python scipy^93^ module. L1000 data includes fluorescence values of several replicas for transcriptional measurements of both drug-treated samples and DMSO treated samples as control. These two conditions for each landmark (genes directly measured by L1000 assay) gene were compared with one way-Anova method after verifying the assumptions and *p* values for each landmark gene are collected. The *p* values for each gene as drug-treated condition compared against control condition is generated, genes that have lower *p* value than the selected threshold were collected and labeled as “significantly transcribed genes”. These “significantly transcribed genes” are listed for each cell line-drug pair. Also, log_2_ fold changes of average transcription values of each gene compared to the control condition are calculated and stored to be later used in the prize designation of terminals.

From L1000 data, by using the “significantly transcribed genes” list and transcription factor regulatory network^94^, we found out transcription factors that are regulating any of the genes in our list. If a transcription factor regulates at least three of the genes in the significant gene list, we used it as a terminal, and mean log_2_ fold changes of interactors of these transcription factors are used as their prizes. From P100 data, “significantly phosphorylated proteins” are collected using the phosphosites passing the *p* value threshold (*p* < 0.05).

Lastly, these two lists were joined together, prioritizing those coming from proteomic data. Also, the targets of the drug of interest were appended to the terminal set with a uniform weight inferred from the overall weight distribution of all proteins.

### Application of link prediction strategy on the interactome

Link prediction is another step of the input file preparation. Firstly, interactome was processed to exclude hub proteins that have degree >900 and more than ten standard deviations away from the mean (“*UBC*”, “*APP*”, “*ELAVL1*”, “*SUMO2*“, “*CUL3*”) as defined in ref. ^95^. These hub proteins constitute 13,738 interactions in the reference interactome. All edges that include at least one of these hub proteins were excluded. After, the interactome was processed for each drug treatment in which the edges having at least one protein with transcription level, fluorescence measurement, below a threshold were excluded. L1000 data have expression values for each gene that is inferred from direct measurements of landmark genes. For each drug treatment, expression values lie between 0.0 and 15.0. The threshold was set as 2.0, and edges with at least one protein whose expression value is below 2.0 were excluded. iRefWeb interactome and L1000 genes share 11,002 genes in common. After excluding low expressed proteins, condition-specific interactomes included ~160,800 interactions.

After processing with expression values, the link prediction approach is applied. Adamic/Adar scoring method was selected because this link prediction method is known for its ability to weigh rarer features more heavily and exclude hubs. The scoring formula of Adamic/Adar is as below:

3 $${\mathrm{Score}}\left( {x,y} \right) = \mathop {\sum}\nolimits_{\left( {w\,\in\, {\mathrm{Neighbors}}\left( x \right) \cap {\mathrm{Neighbors}}\left( y \right)} \right)} {\frac{1}{{\log |{\mathrm{Neighbors}}\left( w \right)|}}}$$

Edge predictions were scored based on the processed interactome (interactome without hub proteins and low expressed genes). Then the same number of best scoring predictions as the edges found in the processed interactome were taken for further filtering based on subcellular location information. Localization information was gathered from the Human Protein Atlas. Using this information, predictions in which two proteins that do not have any common location were filtered out. The predictions in which at least one of the proteins do not have any localization information were collected since it is currently unknown and it has a possibility to be found in the same location with other protein and the predictions in which two proteins having at least one common location were also included in the artificial edge list. After filtering according to the subcellular localization, the remaining predicted edges were appended to the processed interactome by scaling their scores between 0.0 and 0.5. Their scaled scores were assigned as edge weight.

### Comparison of reconstructed networks

#### Topology-based comparison

Menche et al., 2015 reported a pairwise network similarity scoring method based on mapping drug networks on the interactome and scoring their overlap^50^. They calculate the relative distances of two networks in the interactome. The separation score is measured based on the below formula:

4 $$sAB \equiv \left\langle {dAB} \right\rangle - \frac{{\left\langle {dAA} \right\rangle + \left\langle {dBB} \right\rangle }}{2}$$

Where s_AB_ is the separation score of networks A and B, d_AB_ is the shortest distances between A-B proteins, and d_AA_ and d_BB_ are the shortest distances between proteins within A and B, respectively. If a protein is common between both networks (A and B), the distance d_AB_ of that protein is 0.

For our comparison purposes, this separation scoring method is used. Several separation score matrices were prepared to be able to compare drug networks on different levels. As there are different cell lines, there are matrices for each cell line that effects of drug treatments were compared within a cell type. Separation score matrices for each drug were also prepared to compare the drug’s effect based on the cell type. Finally, a separation score matrix containing scores of pairwise network comparisons of all cell line-drug pair combinations was prepared to have a broader idea. All matrices are subjected to hierarchical clustering using python scipy.cluster.hierarchy module.

#### Pathway-based comparison

Reconstructed networks are subjected to functional analyses based on their KEGG pathway enrichments. Pathway enrichments are calculated using DAVID source code^96^. All significantly enriched pathways (*p* < 0.05) are collected for each network and signaling pathways are filtered to be later used for comparison purposes.

#### Calculation of topological features of the reconstructed networks

The number of nodes, number of edges, average degree, average shortest path lengths, density, and diameter of the networks are calculated with python using the Networkx^97^ module.

#### Hypergeometric tests

Hypergeometric tests are used to analyze the significance of the network overlaps. For this purpose, hypergeom method of python scipy.stats module is used. The parameters of this method are x, M, n, N that are defined as the number of nodes common in both networks, number of nodes in the larger network, number of nodes in the smaller network, and number of total nodes found in the human interactome, respectively.

## Supplementary information

Supplementary Material

Dataset 3

Dataset 1

Dataset 2

Dataset 4

Dataset 5

## Data Availability

All datasets used in this work are publicly available from the following sources: The CMap data was downloaded using accession code GSE101406. Installation details and documentation for Omics Integrator software can be found on this link: https://github.com/fraenkel-lab/OmicsIntegrator. We used the PPI network from https://github.com/fraenkel-lab/OmicsIntegrator (IRefIndex Version 13.0), and drug-target data is curated from Cmap Drug Repurposing Tool (https://clue.io/repurposing-app). The script for parameter tuning performed for network reconstruction can be downloaded from https://github.com/gungorbudak/forest-tuner. The separation score method is adapted from the source code provided in the supplementary material of the study of Menche et al., 2015^50^ (http://science.sciencemag.org/content/suppl/2015/02/18/347.6224.1257601.DC1).

## Code availability

All code used to conduct this study can be found from the GitHub repository on this link: https://github.com/metunetlab/IntegrativeNetworkModeling.
