# Supplementary material for: Functional stratification of cancer drugs through integrated network similarity
Source: NPJ Syst Biol Appl. 2022 Apr 19;8:11. doi: 10.1038/s41540-022-00219-8 (PMC9018743; doi:10.1038/s41540-022-00219-8)
Supplement: Supplementary file 1 — Supplementary Material [file 41540_2022_219_MOESM1_ESM.pdf]

Supplementary Information

Supplementary Note 1

Cancer Drug Categories Differ Based on Chemical Structure and Mechanism of Action.

We started our analysis with the cancer drug classes obtained from their chemical structure and mechanism of action. For this purpose, we used three metrics; i. Tanimoto Similarities calculated with SMILES signatures, ii. MACCS (Molecular ACCess System) keys (166-bit 2D structural fingerprints) distances and iii. Literature-curated mechanisms of action (MoAs) of 70 drugs. The first is pairwise calculations of Jaccard’s coefficient between fingerprints of two compounds[1]; the second is a SMARTS (SMILES arbitrary target specification)-based implementation of the 166 public MACCS keys, which are sometimes referred to as the MDL keys[2].

We constructed a distance matrix of 70 protein-targeting small molecules, including pairwise comparisons based on two metrics. Hierarchical clustering is applied to two distance matrices, and dendrograms are used to infer the most appropriate number of clusters. As each line's height in the dendrograms shows the relative distances between drugs, slicing the dendrogram horizontally at a defined height would provide us the number of clusters. We defined this height to have a balanced level of both the granularity and size of clusters. Dendrogram of Tanimoto distance matrix gave very small groups that only consist of either identical chemical structures or drugs derived from the chemical structure in the same group (Figure S1A) while dendrogram of MACCS keys distance matrix provided five drug clusters (with a cut at the height of 160) in different sizes ranging from one drug to 47 drugs (Figure S1B, Data S5). The size two to three compound groups from Tanimoto distance are also present together in the MACCS groups.

MACCS keys-based fingerprints perform well in the classification of the drug analogs, a.k.a. similar chemical structures. However, many drug analogs are developed according to the biologically active form or bio-activation process of interest[3]. Chemically different compounds in their inactive state can change their conformation and become structurally similar in their active state. Additionally, some compounds have molecular modification context-dependently to increase either the lipid or the water solubility. Despite preserving the compound pharmacophore, the structural distance calculations may be affected by the chemical modifications, and biologically similar drugs may be located in different groups. Given the complexity of drug design and optimization approaches, direct compound classifications based only on the structure may not correctly reflect the biological similarity and mechanism of action. We need to note that we used the open-source MACCS key fingerprints. The higher resolution version of MACCS keys (larger dataset) may give better compound groups than the groups in our analysis.

MoA-based classification of 70 drugs has three clusters, namely kinase inhibitors, epigenetic modulators, and others. Each cluster has subcategories such that kinase inhibitors include tyrosine kinases, dual-specificity kinases, serine/threonine kinases, and other protein kinases (Data S5). An abstract view on 70 compounds in our analysis is illustrated in Figure S2 based on their main- and subcategories and MoA. The number of drugs in clusters of MoA are generally balanced (each MoA has 1-3 drugs). However, some clusters have more drugs, such as HDAC inhibitors, histone lysine methylase inhibitors, CDK inhibitors, and most of the clusters in the other category have only one drug. Drugs included in the same MACCS cluster have diverging MoAs such that everolimus and sirolimus target protein kinases (mTOR inhibitors), tacrolimus targets a phosphatase (calcineurin inhibitor); dexamethasone, verteporfin, pravastatin, and geldanamycin have also different MoAs. On the other hand, HDAC inhibitors

are separated into two different clusters according to their MACCS key distances, though they have similar MoA.

Supplementary Note 2

Topological Analysis of the Reconstructed Networks

Topological properties of cell line-drug networks revealed that larger networks are observed in the MCF7 cell line, and the size of the network depends on the centrality of the drug targets. Moreover, the most frequently found 100 proteins in networks display essential roles in several cancer-related signaling pathways, biological and molecular processes.

We could model 70 drug networks for A375, 46 drug networks for A549, 43 drug networks for MCF7, 59 drug networks for PC3, and 18 drug networks for YAPC.

The comparison dependent on node frequency gave a merged set of 1908 proteins across all networks. The most frequently present 100 proteins are enriched in MAPK signaling pathway, AMPK signaling pathway, mTOR signaling pathway, PPAR signaling pathway, insulin signaling pathway, cell cycle, and some other cancer-related pathways. These results are consistent with the known active signaling pathways in cancer cells. For example, the PPAR signaling pathway is related to fatty acid metabolism. Cancer cells need to produce more energy, so the fatty acid degradation process is activated. Insulin signaling is also related to the energy needs of the cells. Additionally, we observed that transcription regulation, DNA-binding, and protein heterodimerization activity are mostly active functions (Figure S3).

The number of nodes, number of edges, average degree, average shortest path lengths, density, and diameter of networks in each cell line are summarized in FigureS3. We observed larger networks in the MCF7 cell line such that the number of nodes ranges between 28 and 252, and 60% of drug networks in MCF7 have more than 100 nodes. Networks of A375 and

PC3 cell lines also have a high number of nodes (A375: 14-196 nodes, PC3: 23-207). We checked the degrees of drug targets in large networks if they constitute hubs in networks. Some of the HDAC inhibitors, e.g., Belinostat, Entinostat, Trichostatin-a, usually have large networks, and HDAC1, one of their targets, has a high degree compared to other targets.

Moreover, LY-294002, a broad-spectrum drug with several target proteins, has large networks such that the number of nodes in A375, MCF7, PC3, and YAPC are more than 100 nodes, and the number of nodes in the A549 network is 95. One of the targets of LY-294002 is MAPK1, and it usually has a high degree. The average shortest path lengths of YAPC are higher than other cell lines, while A549 is the cell line with the lowest average number of nodes and edges (Figure S4).

We observed that as the network gets larger, it is more probable to cover several cancer-related signaling pathways and higher overlap. On the other hand, when at least one of the networks is small (number of nodes<100), the network pair is more prone to be separated. If one drug alters many omic entities in a cell line, it may modulate multiple pathways, resulting in a large network. On the other hand, another drug may modulate a small network comprising only one or two pathways. The former is a more generalist drug, and the latter is a more specialist drug. We note that the separation scores of two large networks are significantly smaller than the ones between two small networks. This result is expected because the probability of having overlap between generalist drugs is higher than the specialists. To test the significance of the overlap between two networks, we applied the hypergeometric test. We found that the negative separation scores imply significant overlap (p-value < 0.05) (Data S1, Figure S5).

Supplementary Note 3

Analysis of separation score calculation in terms of the difference between our network-based approach against the list of seed proteins

The comparison of the cell line–drug conditions is mainly performed by the measure of separation score proposed by Menche et al.,2015. Since this method only requires two sets of genes/proteins. Two application of this method is performed. First, the method is directly applied to seed proteins collected from transcriptomic and phosphoproteomic data and drug targets. Second, pairwise separation scores are calculated for the reconstructed networks. The difference between two inputs, seed proteins and network nodes for a given condition, would be that a subset of seed proteins may not be found within the reconstructed networks. There may be additional proteins in the set of network nodes since the network reconstruction method used in this study, Omics Integrator, is able to find Steiner nodes that may not be identified experimentally. These kinds of differences may lead to different scores between conditions. Two separation score matrices produced by two approaches are shown in Figure S6. Our network-based approach provided a small subset of the cell line–drug conditions with negative separation scores (overlapping networks) (Figure S6A). However, lists of seed proteins constitute generally similar modules such that most of the separation scores lie in the negative range meaning higher similarity (Figure S6B). Because of molecular heterogeneity, any drugs are rarely expected to similarly modulate the same pathways in all cancer types, implying false positives across cancer types.

The difference between two applications of separation score distributions, direct and network-based application, stems mainly from the intermediate (Steiner) nodes found via the network reconstruction method. These intermediate proteins can potentially reveal the off-target effects of the drugs. Additionally, the spurious and low-confidence interactions within the reference interactome may be used to find the shortest paths between the seed proteins in the direct

application of the method. On the other hand, the PCSF results provide an optimal network with high confidence interactions. Thus, the overlap between drugs with different MoA is higher in the direct comparison than the network-based comparison.

Consequently, the false positive rate is significantly higher in the direct application of Menche's method compared to the network-based application. The best performance of the network-based classification was achieved with the separation score threshold of -0.45 (MCC=0.192 and FPR=0.083). When the same threshold is used for the direct application to the seed proteins without network reconstruction, the performance is significantly low with many false positives (MCC=0.116 and FPR=0.358). As a result, integrative network modeling provides more stringent and biologically relevant results.

Supplementary Note 4

Analysis of the effect of link prediction on the reconstruction networks

We calculated the percentages of predicted edge numbers in the networks to understand the effect of link prediction on network reconstruction. The average percentage of predicted edge presence is only 3.2%, and cell line-based averages range between 2.7%(MCF7) and 4.2%(A549). The percentage of predicted edge presence in individual networks does not exceed 12% of the whole network (for example, in the PC3-roscovitine network, 11.8% of edges are predicted edges, and in the A549-methylstat network, 11.5% of edges are predicted edges.) (Supplementary Table 1 and Data S2). Moreover, we collected all predicted edges observed in all networks and confirmed if they were found in the STRING55 database. Out of 545 predicted edges, 180 (33%) are cataloged in STRING with combined scores ranging between 0.403 and 0.999. We investigated these predicted edges to understand if they cause Omics Integrator to select unnecessary Steiner nodes while modeling networks and if our network comparison

approach is affected by the existence of predicted edges. This analysis revealed that link prediction does not cause any misleading outcomes for two reasons: (i) there is no additional node in networks connected with only predicted edges, (ii) there is no predicted edge in the reference interactome used for separation score calculation.

Predicted edges that are appended to the edges in the human interactome can display both false positive and false negative interactions. For the former case, these predicted edges can be considered as noise and expose the robustness of the reconstructed networks. If the addition of noise to the interactome results as reconstructed networks with a high percentage of predicted edges, this would mean low robustness. On the contrary, a low percentage of predicted edges would indicate that reconstructed networks are more dependent on the omic data and less prone to noise. For the latter case; that is, the predicted edges are false negative edges of the human interactome, these interactions will be important to study in specific cell line-drug conditions. They can be considered as new candidate interactions in experimental protocols.

Supplementary Figures

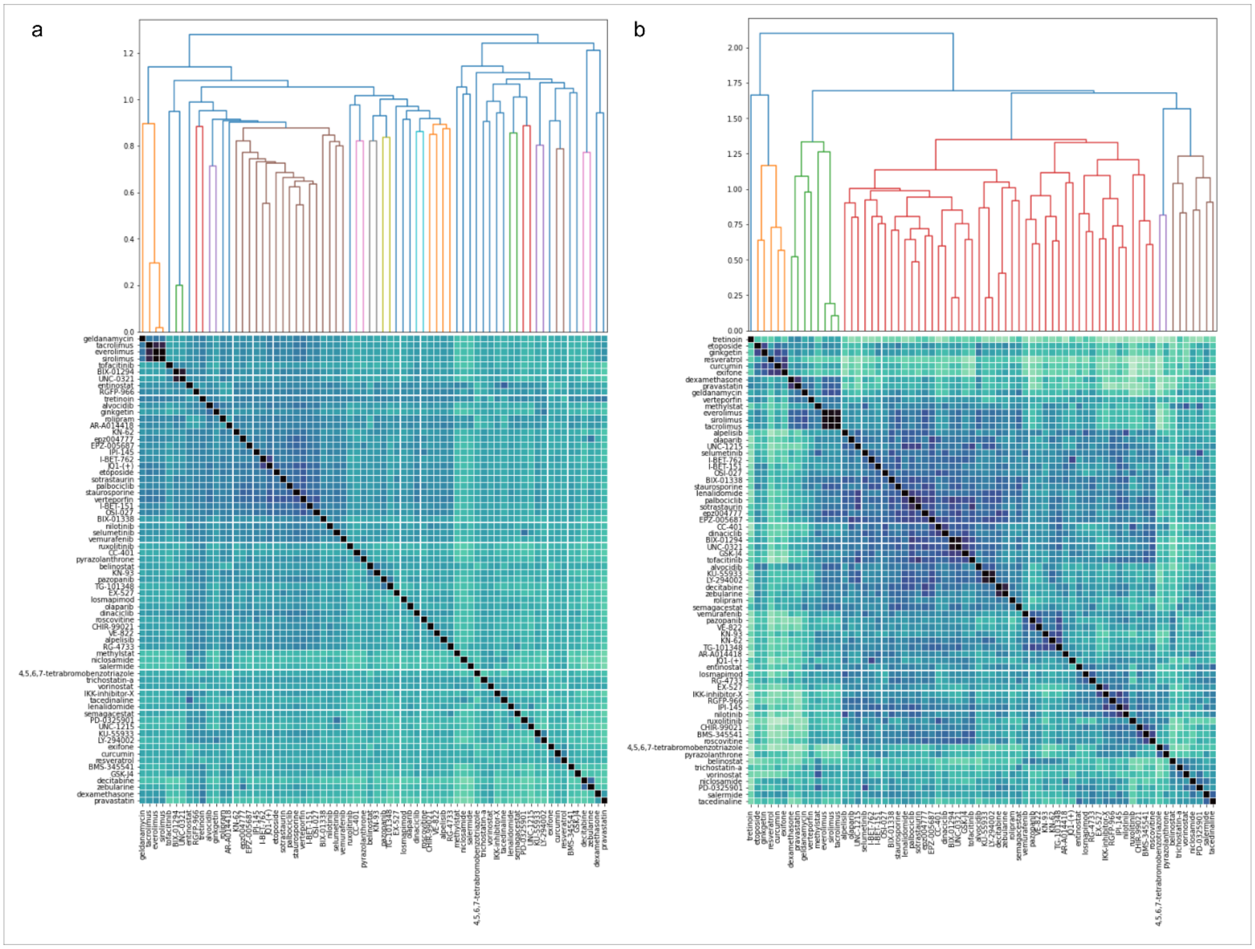

Supplementary Figure 1. Chemical similarity matrices of 70 drugs. a) Tanimoto Similarities – Hierarchically clustered based on Euclidean Distance **b)** MACCS key distance similarities – Hierarchically clustered based on Euclidean Distance

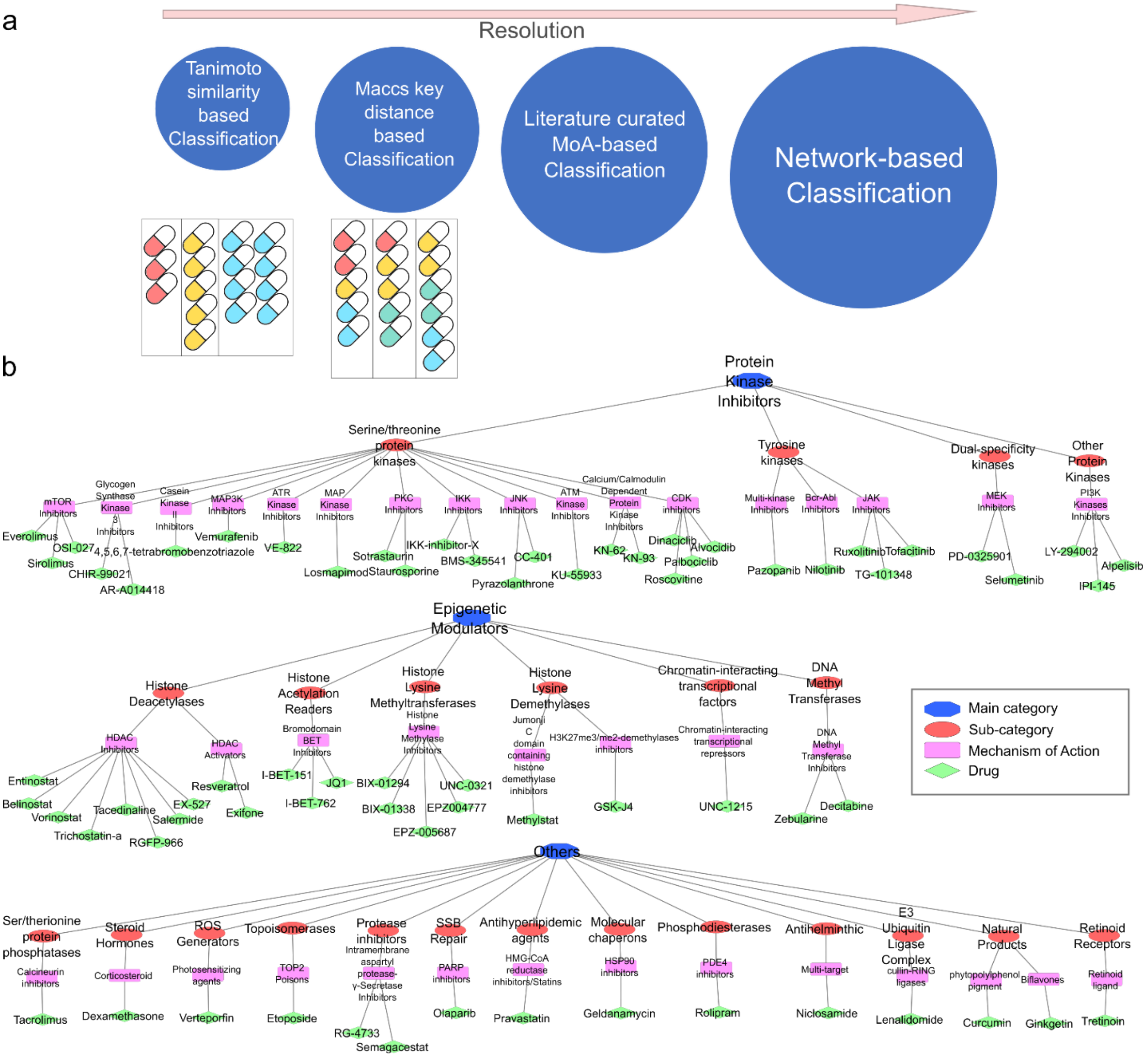

Supplementary Figure 2. Representation of drug classification procedures. a) Resolution increases towards network-based classification. Tanimoto similarity-based classification cannot discriminate drug classes adequately such that drugs either split into clusters with 2-3 drugs or clusters with large numbers of elements. Moving further with MACCS key distance-based classification and literature curated MoA-based classification, the resolution gets better. Still, drug clusters of the former and the latter may be different and cannot appropriately reflect biological relevance. Network-based drug classification produced drug clusters that apply to specific cell types and conditions. b) Drugs that are classified according to prior Mechanism of

Action (MoA) knowledge. There are three main categories; protein kinases, epigenetic modulators, and others. Main categories are divided into subcategories that reflect the general biological functions. Each subcategory includes the specific MoA of drugs.

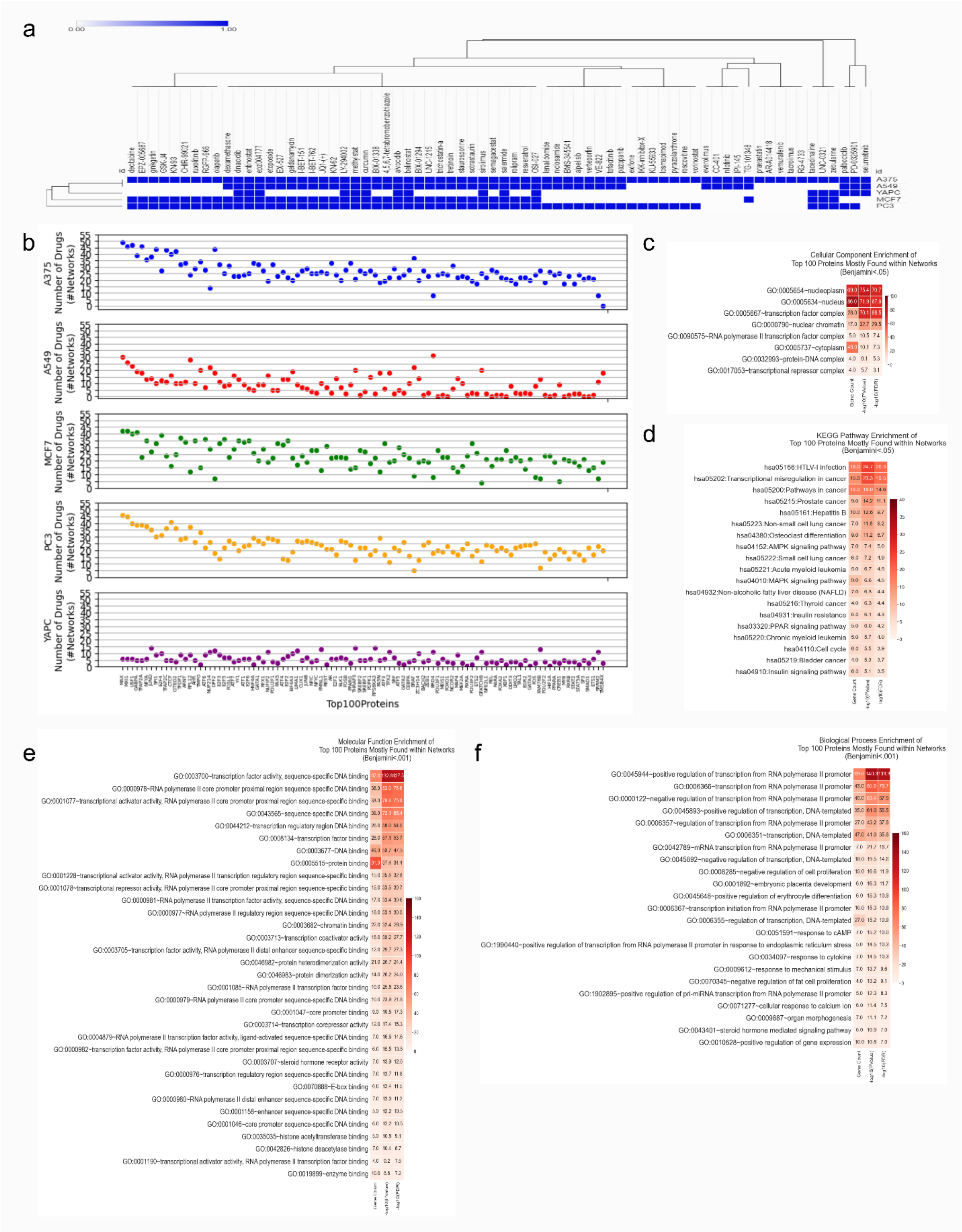
a
b
A375 Number of Drugs (#Networks)
A549 Number of Drugs (#Networks)
MCF7 Number of Drugs (#Networks)
PC3 Number of Drugs (#Networks)
YAPC Number of Drugs (#Networks)
Top100Proteins
c
Cellular Component Enrichment of Top 100 Proteins Mostly Found within Networks (Benjamini<.05)
GO:0005654~nucleoplasm
GO:0005634~nucleus
GO:0005667~transcription factor complex
GO:0000790~nuclear chromatin
GO:0090575~RNA polymerase II transcription factor complex
GO:0005737~cytoplasm
GO:0032993~protein-DNA complex
GO:0017053~transcriptional repressor complex
d
KEGG Pathway Enrichment of Top 100 Proteins Mostly Found within Networks (Benjamini<.05)
hsa05166:HTLV-I infection
hsa05202:Transcriptional misregulation in cancer
hsa05200:Pathways in cancer
hsa05215:Prostate cancer
hsa05161:Hepatitis B
hsa05223:Non-small cell lung cancer
hsa04380:Osteoclast differentiation
hsa04152:AMPK signaling pathway
hsa05222:Small cell lung cancer
hsa05221:Acute myeloid leukemia
hsa04010:MAPK signaling pathway
hsa04932:Non-alcoholic fatty liver disease (NAFLD)
hsa05216:Thyroid cancer
hsa04931:Insulin resistance
hsa03320:PPAR signaling pathway
hsa05220:Chronic myeloid leukemia
hsa04110:Cell cycle
hsa05219:Bladder cancer
hsa04910:Insulin signaling pathway
e
Molecular Function Enrichment of Top 100 Proteins Mostly Found within Networks (Benjamini<.001)
f
Biological Process Enrichment of Top 100 Proteins Mostly Found within Networks (Benjamini<.001)
GO:0045944~positive regulation of transcription from RNA polymerase II promoter
GO:0006366~transcription from RNA polymerase II promoter
GO:0000122~negative regulation of transcription from RNA polymerase II promoter
GO:0045893~positive regulation of transcription, DNA-templated
GO:0006357~regulation of transcription from RNA polymerase II promoter
GO:0006351~transcription, DNA-templated
GO:0042789~mRNA transcription from RNA polymerase II promoter
GO:0045892~negative regulation of transcription, DNA-templated
GO:0008285~negative regulation of cell proliferation
GO:0001892~embryonic placenta development
GO:0045648~positive regulation of erythrocyte differentiation
GO:0006367~transcription initiation from RNA polymerase II promoter
GO:0006355~regulation of transcription, DNA-templated
GO:0051591~response to cAMP
GO:1990440~positive regulation of transcription from RNA polymerase II promoter in response to endoplasmic reticulum stress
GO:0034097~response to cytokine
GO:0009612~response to mechanical stimulus
GO:0070345~negative regulation of fat cell proliferation
GO:1902895~positive regulation of pri-miRNA transcription from RNA polymerase II promoter
GO:0071277~cellular response to calcium ion
GO:0009887~organ morphogenesis
GO:0043401~steroid hormone mediated signaling pathway
GO:0010628~positive regulation of gene expression

Supplementary Figure 3. General analysis of most frequently found 100 proteins in 236 cell line-drug networks. a) Heatmap showing the network availability of each cell line and drug condition. Blue boxes refer to the existence of reconstructed networks. **b)** Number of networks per cell line in which the most frequently found 100 proteins have appeared. **c-f)** Functional enrichments of top 100 proteins; cellular component, Kegg pathways, molecular function, and biological function.

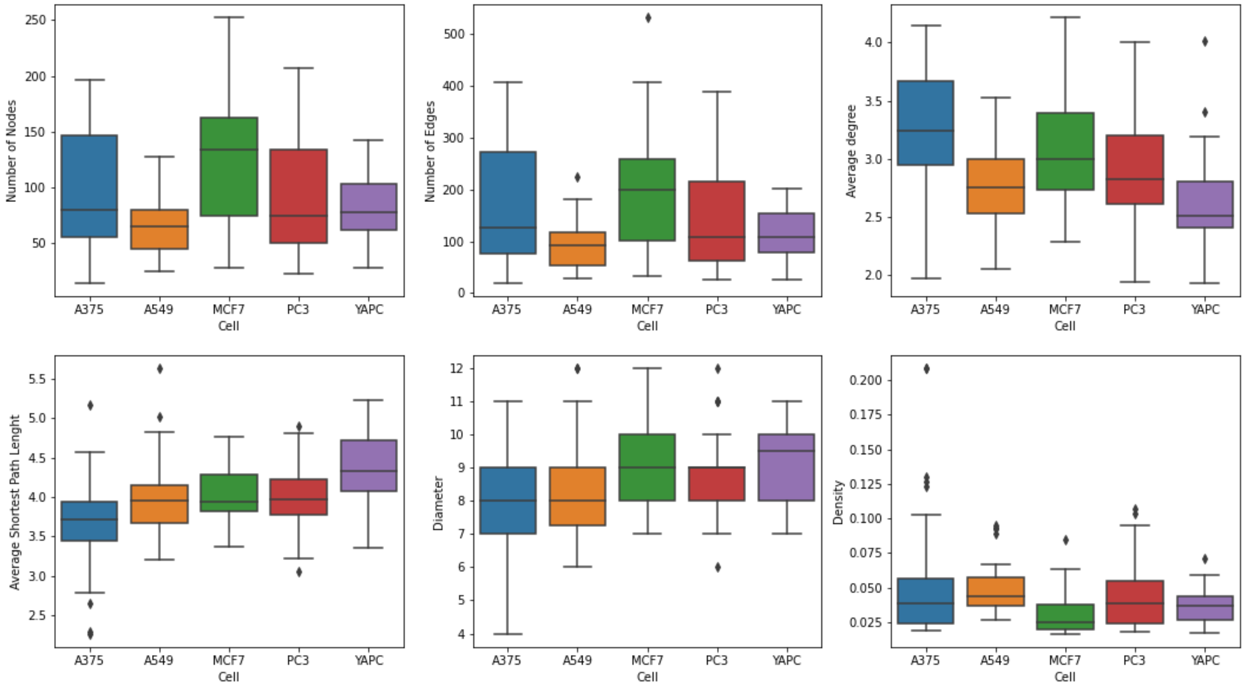

Supplementary Figure 4. Topological properties of networks reconstructed for five cell lines

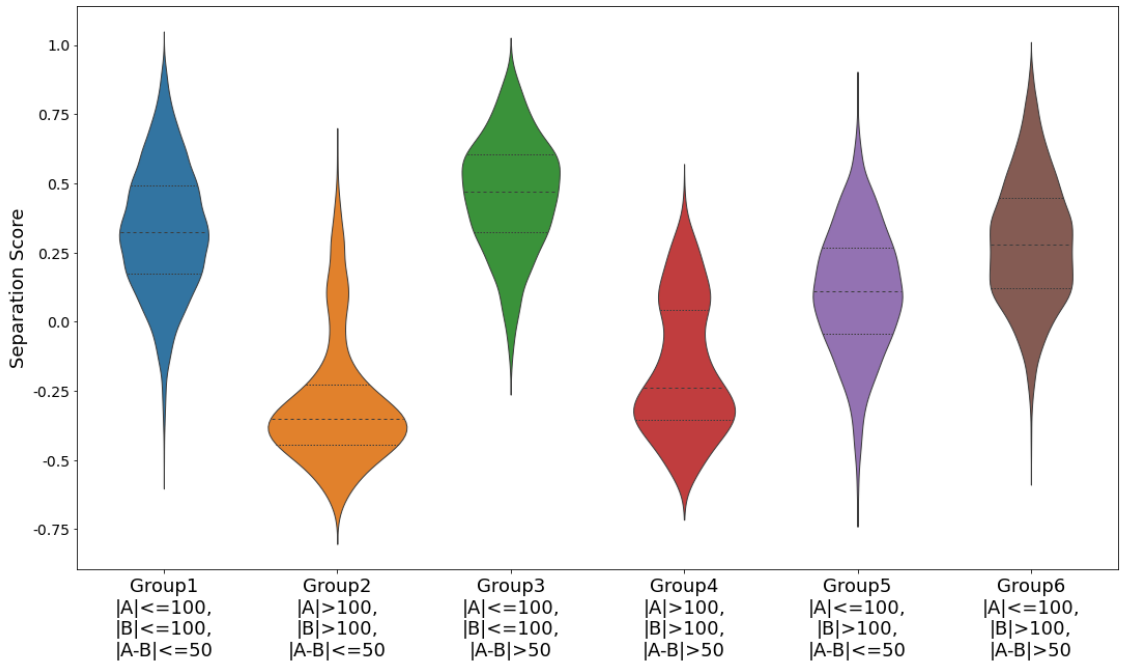

Supplementary Figure 5. Distribution of separation scores based on the types of network pairs.

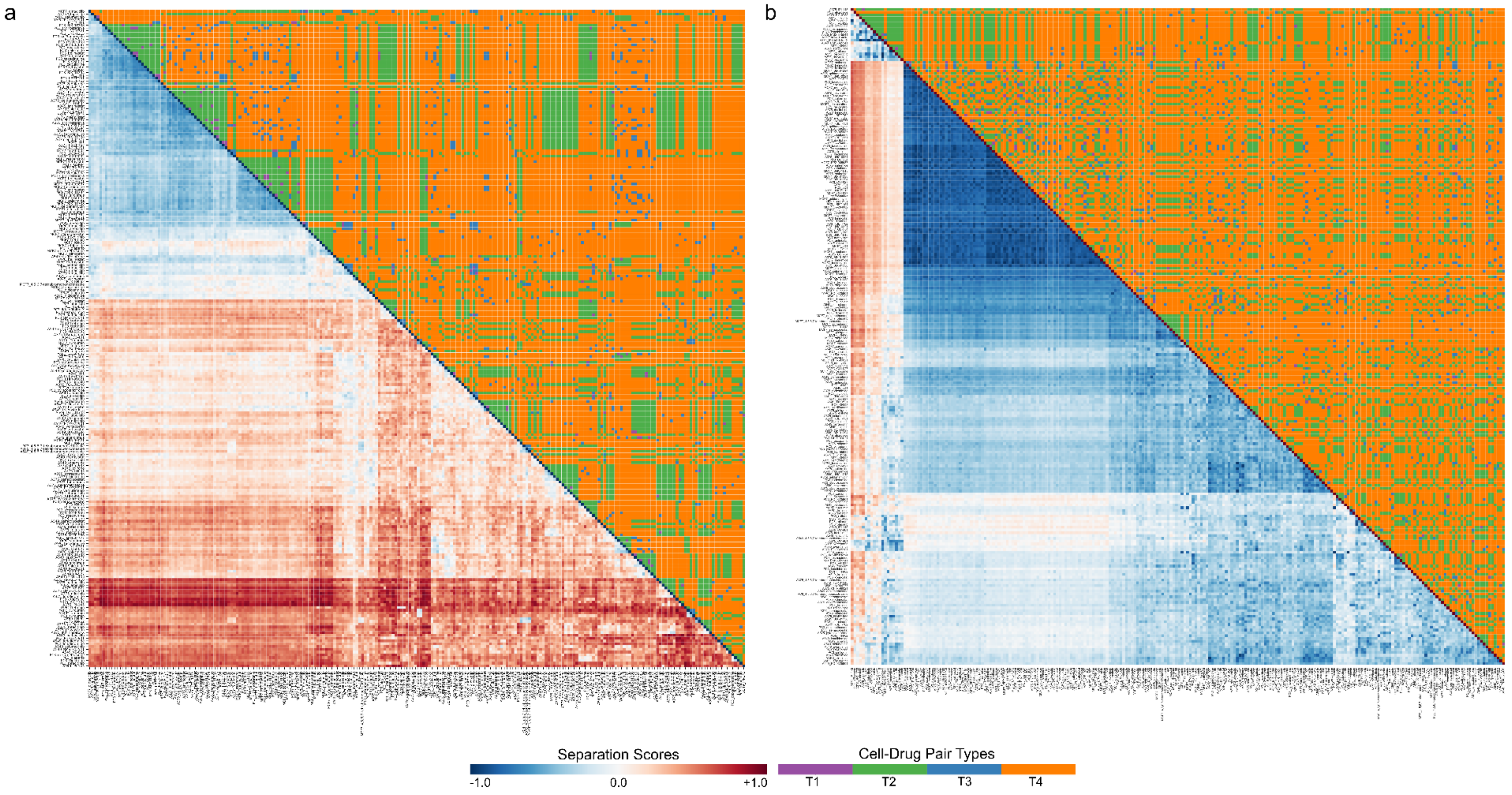

Supplementary Figure 6. Separation score matrices depicting comparison between 236 cell line–drug conditions. a) Separation score matrix of reconstructed networks **b)** Separation score matrix of seed protein lists

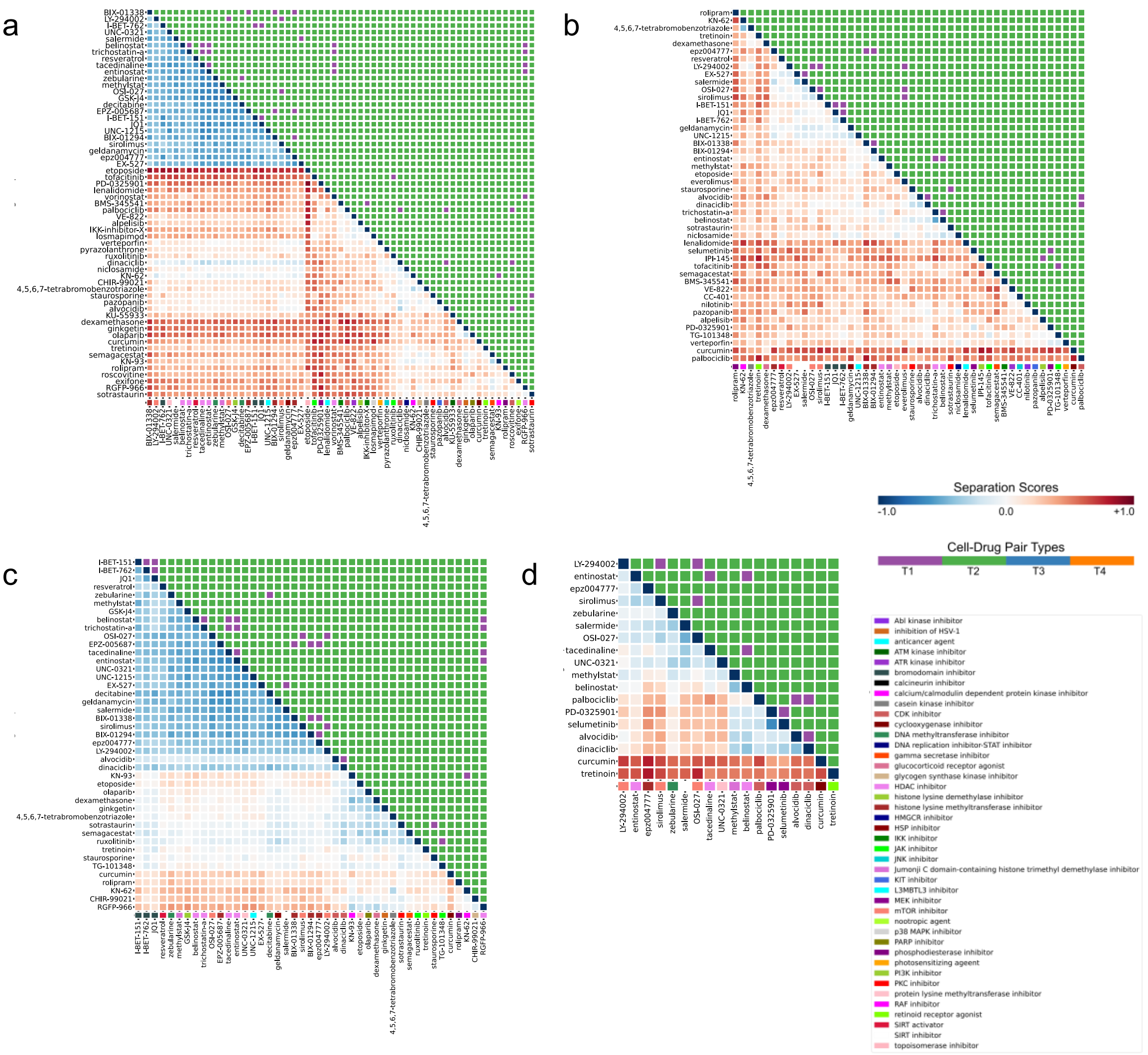

Supplementary Figure 7. Cell line-based network separation score matrices. a) PC3 **b)** A549 **c)** MCF7 **d)** YAPC. Drugs are clustered based on the separation scores, and color keys reflect s_{AB}. The upper triangle of the heatmap highlights the four distinct classes defined based on the cell line and MoA types (T1: same cell types and MoAs; T2: same cell types but different MoAs; T3: different cell types but the same MoAs; T4: different cell types and MoAs). Colors on the x-axis refer to the MoA of each column mapped on the right of the heatmap.

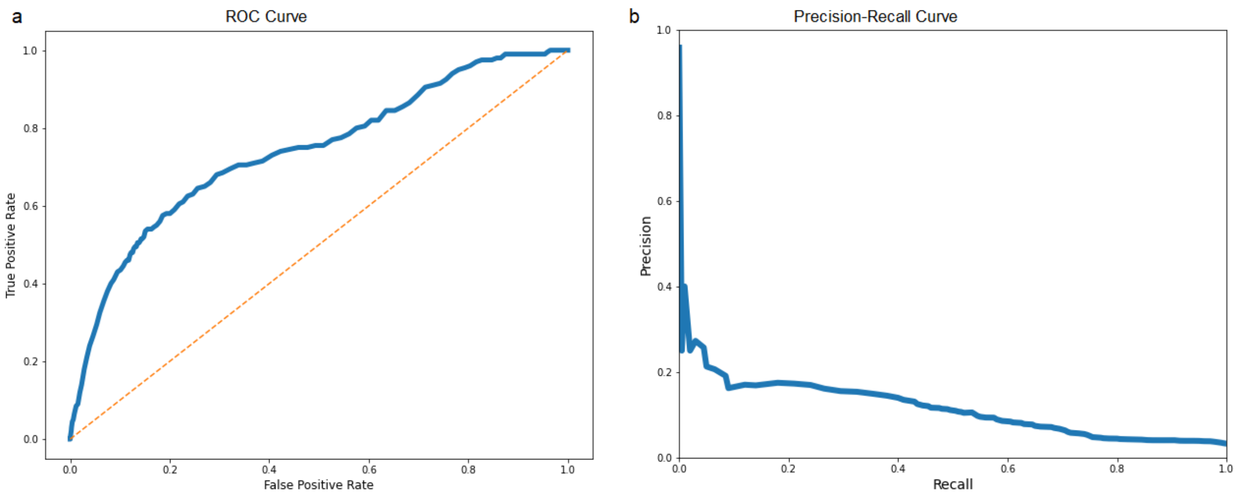

Supplementary Figure 8. a) ROC Curve **b)** Precision-Recall Curve using the ground truth as the drugs with the same MoA would have similar networks and the drugs with different MoA would have different networks.

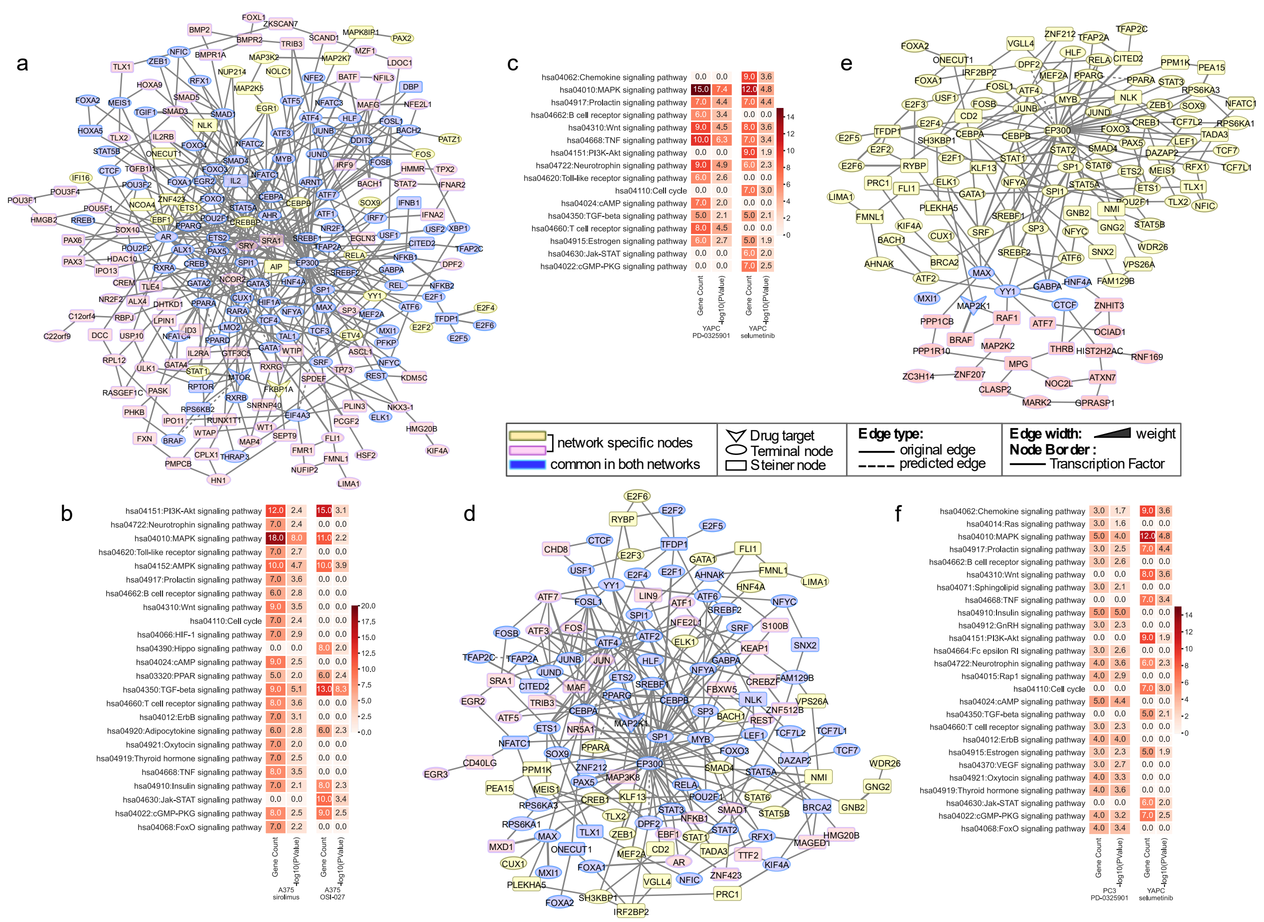

Supplementary Figure 9. Pairwise network comparisons for mTOR inhibitors and MEK inhibitors. a-b) Merged network maps of sirolimus and OSI-027 in A375 cell line (a) and signaling pathways enriched in their networks (b). **c-d)** Merged network maps of PD-0325901 and selumetinib in YAPC cell line (c) and signaling pathways enriched in their networks (d). **e-f)** Merged network maps of PD-0325901 in PC3 cell line and selumetinib in YAPC cell line (e) and signaling pathways enriched in their networks (f). Proteins common in both networks are shown with blue nodes.

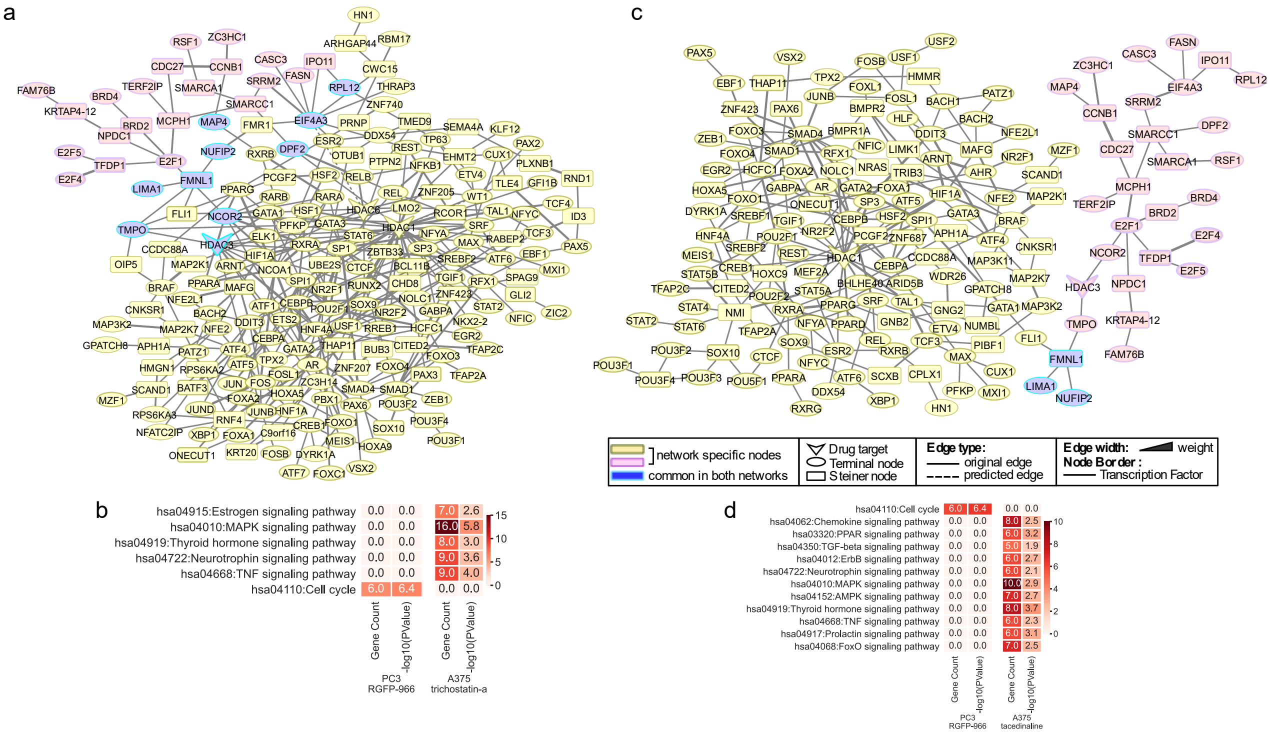

Supplementary Figure 10. Pairwise network comparisons for HDAC inhibitors. a-b) Merged network maps of RGFP-966 in PC3 cell line (pink nodes) and Trichostatin-a in A375 cell line (yellow nodes) (a) and signaling pathways enriched in their networks (b). **c-d)** Merged network maps of RGFP-966 in PC3 cell line (pink nodes) and Tacedinaline in A375 cell line(yellow nodes) (c) and signaling pathways enriched in their networks (d). Proteins common in both networks are shown with blue nodes.

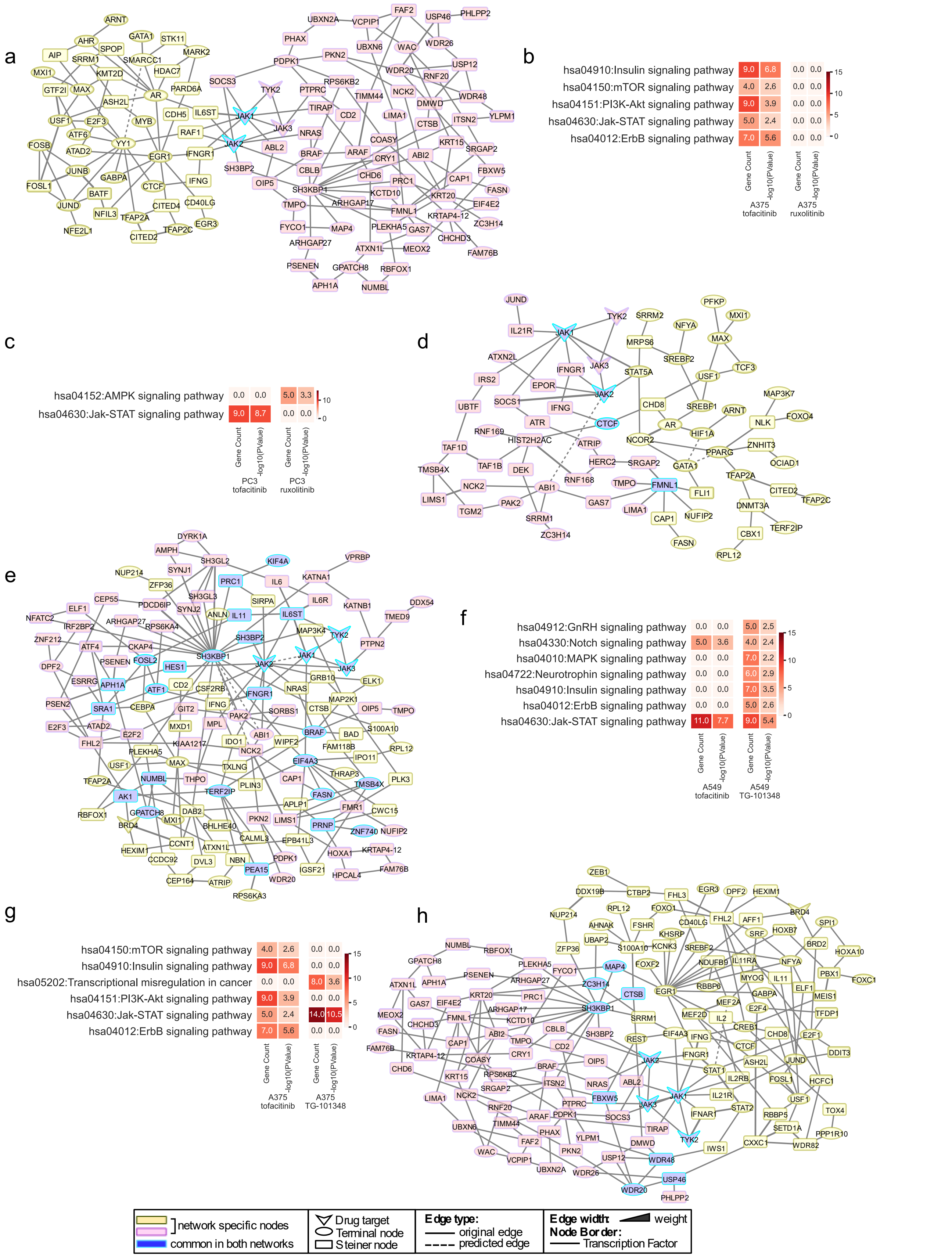

a
b
hsa04910:Insulin signaling pathway 9.0 6.8 0.0 0.0
hsa04150:mTOR signaling pathway 4.0 2.6 0.0 0.0
hsa04151:PI3K-Akt signaling pathway 9.0 3.9 0.0 0.0
hsa04630:Jak-STAT signaling pathway 5.0 2.4 0.0 0.0
hsa04012:ErbB signaling pathway 7.0 5.6 0.0 0.0
Gene Count
-log10(PValue)
A375 tofacitinib
A375 ruxolitinib
c
hsa04152:AMPK signaling pathway 0.0 0.0 5.0 3.3
hsa04630:Jak-STAT signaling pathway 9.0 8.7 0.0 0.0
PC3 tofacitinib
PC3 ruxolitinib
d
e
f
hsa04912:GnRH signaling pathway 0.0 0.0 5.0 2.5
hsa04330:Notch signaling pathway 5.0 3.6 4.0 2.4
hsa04010:MAPK signaling pathway 0.0 0.0 7.0 2.2
hsa04722:Neurotrophin signaling pathway 0.0 0.0 6.0 2.9
hsa04910:Insulin signaling pathway 0.0 0.0 7.0 3.5
hsa04012:ErbB signaling pathway 0.0 0.0 5.0 2.6
hsa04630:Jak-STAT signaling pathway 11.0 7.7 9.0 5.4
A549 tofacitinib
A549 TG-101348
g
hsa04150:mTOR signaling pathway 4.0 2.6 0.0 0.0
hsa04910:Insulin signaling pathway 9.0 6.8 0.0 0.0
hsa05202:Transcriptional misregulation in cancer 0.0 0.0 8.0 3.6
hsa04151:PI3K-Akt signaling pathway 9.0 3.9 0.0 0.0
hsa04630:Jak-STAT signaling pathway 5.0 2.4 14.0 10.5
hsa04012:ErbB signaling pathway 7.0 5.6 0.0 0.0
A375 tofacitinib
A375 TG-101348
h
network specific nodes
common in both networks
Drug target
Terminal node
Steiner node
Edge type:
original edge
predicted edge
Edge width: weight
Node Border:
Transcription Factor

Supplementary Figure 11. Pairwise network comparisons for JAK inhibitors. a-b) Merged network maps of tofacitinib and ruxolitinib in A375 cell line (a) and signaling pathways enriched in their networks (b). **c-d)** Merged network maps of tofacitinib and ruxolitinib in the PC3 cell line (c) and signaling pathways enriched in their networks (d). **e-f)** Merged network maps of tofacitinib and TG-101348 in A549 cell line (e) and signaling pathways enriched in their networks (f). **g-h)** Merged network maps of tofacitinib and TG-101348 in A375 cell line (g) and signaling pathways enriched in their networks (h).

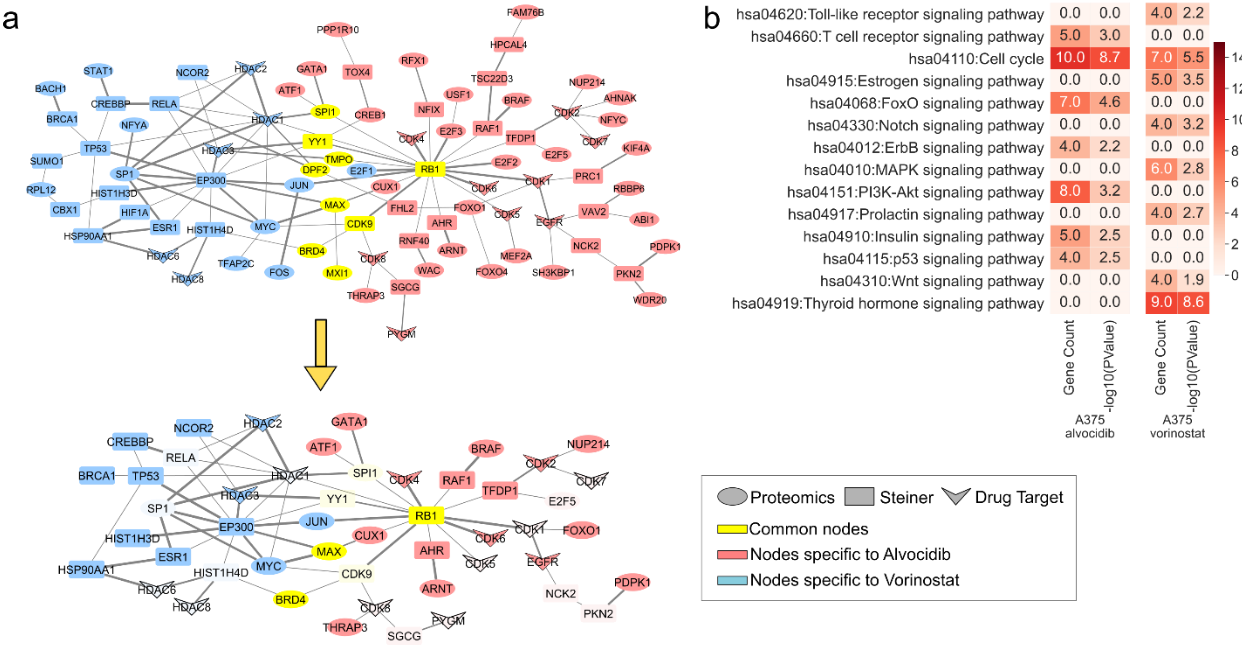

Supplementary Figure 12. Illustration of Alvocidib-Vorinostat combination in A375 in terms of the complementary exposure principle

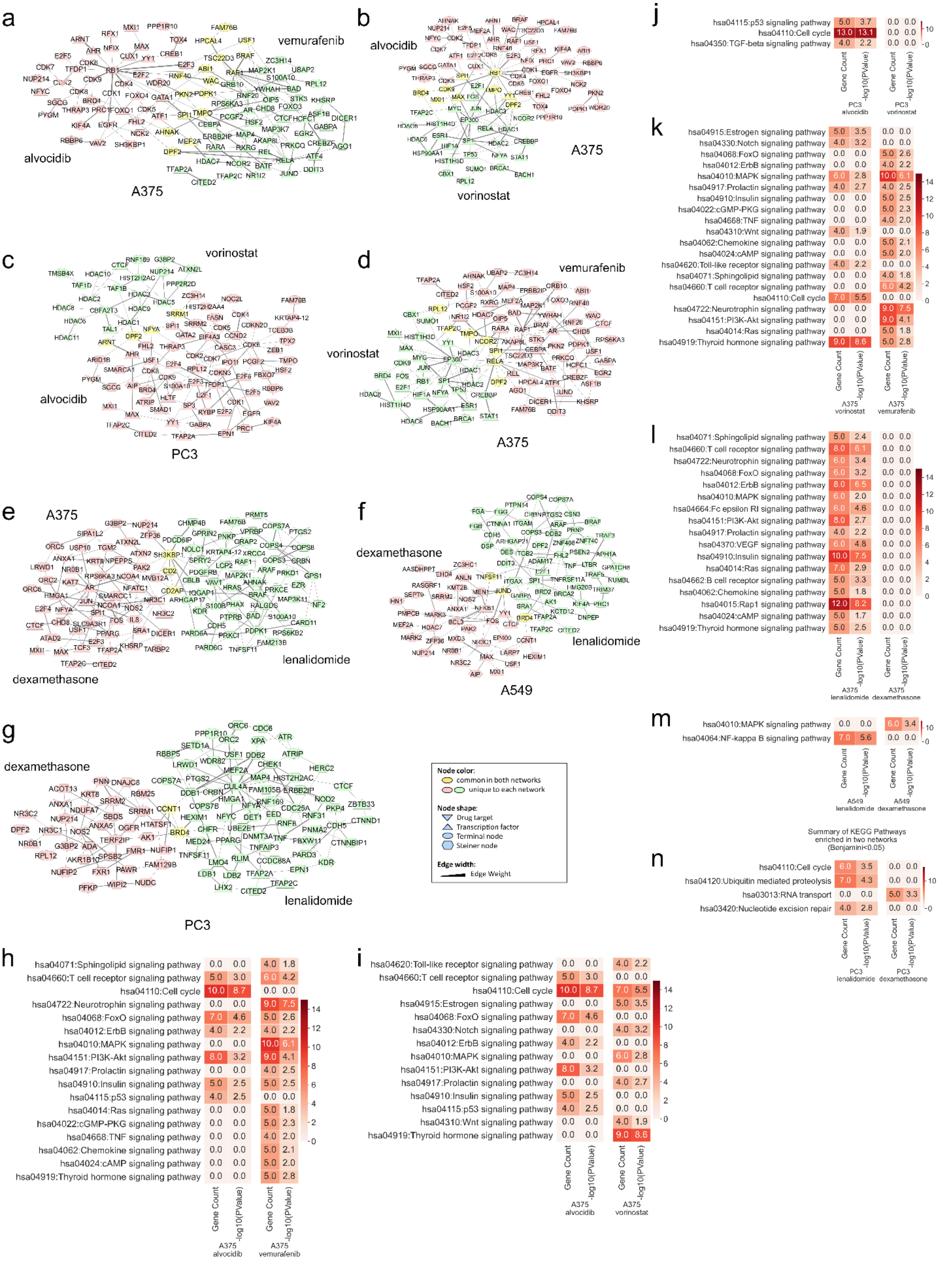
a
vemurafenib
alvocidib
A375
b
alvocidib
A375
vorinostat
c
vorinostat
alvocidib
PC3
d
vemurafenib
vorinostat
A375
e
A375
dexamethasone
lenalidomide
f
dexamethasone
lenalidomide
A549
g
dexamethasone
lenalidomide
PC3
Node color:
common in both networks
unique to each network
Node shape:
Drug target
Transcription factor
Terminal node
Steiner node
Edge width:
Edge Weight
Summary of KEGG Pathways enriched in two networks (Benjamini<0.05)
h
i
j
k
l
m
n

Supplementary Figure 13. Pairwise network (a-g) and signaling pathway (h-n) comparisons for experimentally validated drug pairs used for cancer as a combination. Names of drugs and cell lines are denoted on the figure.

Supplementary Tables

Supplementary Table 1. Percentages of the number of predicted edges found within cell line-drug networks

Cell Line	Max Predicted Edge Frequency within a Network (%)	Average Predicted Edge Frequency for All Networks (%)
A375	11	3
A549	12	4
MCF7	8	2
PC3	12	3
YAPC	12	3

Supplementary Table 2. Drug pairs with different MoA but overlapping networks in A375 with separation scores lower than -0.50.

Drug1	Drug2	Separation Score	Citation
GSK-J4	entinostat	-0.64	[4]
GSK-J4	tacedinaline	-0.59	[4]
GSK-J4	trichostatin-a	-0.57	[4]
zebularine	entinostat	-0.50	[5]
resveratrol	entinostat	-0.70	[6]
belinostat	GSK-J4	-0.67	[4]
decitabine	entinostat	-0.65	[7,8]
geldanamycin	salermide	-0.65	[9]
resveratrol	decitabine	-0.64	[10]
belinostat	decitabine	-0.62	[8,11,12]
sirolimus	resveratrol	-0.59	[13–15]
sirolimus	decitabine	-0.55	[16]
resveratrol	trichostatin-a	-0.53	[6]
resveratrol	tacedinaline	-0.52	[6]
decitabine	salermide	-0.62	[8]
decitabine	EX-527	-0.62	[8]
belinostat	resveratrol	-0.60	[6]
decitabine	tacedinaline	-0.51	[8]
zebularine	GSK-J4	-0.60	[17]
zebularine	I-BET-151	-0.59	[17]
zebularine	BIX-01294	-0.55	[17]
zebularine	JQ1	-0.55	[17]
zebularine	resveratrol	-0.51	[18]

Supplementary References

1. Baldi P, Nasr R. When is chemical similarity significant? The statistical distribution of chemical similarity scores and its extreme values. *J Chem Inf Model*. 2010;50(7):1205-1222. doi:10.1021/ci100010v

2. Durant JL, Leland BA, Henry DR, Nourse JG. Reoptimization of MDL keys for use in drug discovery. *J Chem Inf Comput Sci*. 2002;42(6):1273-1280. doi:10.1021/ci010132r

3. Drug discovery and development. May 2021. https://chem.libretexts.org/@go/page/227708.

4. Kayabolen A, Sahin GN, Seker F, et al. EXTH-10. A COMBINATION OF EPIGENETIC ENZYME INHIBITORS, GSK-J4 AND BELINOSTAT, REVEALS HIGH EFFICACY IN IDH1 MUTANT GLIOMAS. *Neuro Oncol*. 2020;22(Supplement_2):ii88-ii89. doi:10.1093/neuonc/noaa215.364

5. Sanaei M, Kavoosi F. Investigation of the Effect of Zebularine in Comparison to and in Combination with Trichostatin A on p21Cip1/Waf1/ Sdi1, p27Kip1, p57Kip2, DNA Methyltransferases and Histone Deacetylases in Colon Cancer LS 180 Cell Line. *Asian Pacific J Cancer Prev*. 2020;21(6):1819-1828. doi:10.31557/APJCP.2020.21.6.1819

6. Lucas J, Hsieh T-C, Halicka HD, Darzynkiewicz Z, Wu JM. Upregulation of PD-L1 expression by resveratrol and piceatannol in breast and colorectal cancer cells occurs via HDAC3/p300-mediated NF-κB signaling. *Int J Oncol*. 2018;53(4):1469-1480. doi:10.3892/ijo.2018.4512

7. Wang C, Hamacher A, Petzsch P, et al. Combination of Decitabine and Entinostat Synergistically Inhibits Urothelial Bladder Cancer Cells via Activation of FoxO1. *Cancers (Basel)*. 2020;12(2):337. doi:10.3390/cancers12020337

8. Kalac M, Scotto L, Marchi E, et al. HDAC inhibitors and decitabine are highly synergistic and associated with unique gene-expression and epigenetic profiles in models of DLBCL. *Blood*. 2011;118(20):5506-5516. doi:10.1182/blood-2011-02-336891

9. Clark-Knowles K V, He X, Jardine K, et al. Reversible modulation of SIRT1 activity in a mouse strain. *PLoS One*. 2017;12(3):e0173002. https://doi.org/10.1371/journal.pone.0173002.

10. Clouser CL, Chauhan J, Bess MA, et al. Anti-HIV-1 activity of resveratrol derivatives and synergistic inhibition of HIV-1 by the combination of resveratrol and decitabine. *Bioorg Med Chem Lett*. 2012;22(21):6642-6646. doi:10.1016/j.bmcl.2012.08.108

11. Oza J, Lee SM, Weiss MC, et al. A phase 2 study of belinostat and SGI-110 (guadecitabine) for the treatment of unresectable and metastatic conventional chondrosarcoma. *J Clin Oncol*. 2021;39(15_suppl):TPS11578-TPS11578. doi:10.1200/JCO.2021.39.15_suppl.TPS11578

12. Steele N, Finn P, Brown R, Plumb JA. Combined inhibition of DNA methylation and histone acetylation enhances gene re-expression and drug sensitivity in vivo. *Br J Cancer*. 2009;100(5):758-763. doi:10.1038/sj.bjc.6604932

13. Alayev A, Salamon RS, Sun Y, et al. Effects of combining rapamycin and resveratrol on apoptosis and growth of TSC2-deficient xenograft tumors. *Am J Respir Cell Mol Biol*. 2015;53(5):637-646. doi:10.1165/rcmb.2015-0022OC

14. Alayev A, Salamon RS, Schwartz NS, Berman AY, Wiener SL, Holz MK. Combination of Rapamycin and Resveratrol for Treatment of Bladder Cancer. *J Cell Physiol*. 2017;232(2):436-446. doi:https://doi.org/10.1002/jcp.25443

15. Alayev A, Berger SM, Holz MK. Resveratrol as a novel treatment for diseases with mTOR

pathway hyperactivation. *Ann N Y Acad Sci*. 2015;1348(1):116-123. doi:https://doi.org/10.1111/nyas.12829

16. Liesveld JL, O'Dwyer K, Walker A, et al. A phase I study of decitabine and rapamycin in relapsed/refractory AML. *Leuk Res*. 2013;37(12):1622-1627. doi:10.1016/j.leukres.2013.09.002

17. Sdelci S, Lardeau C-H, Tallant C, et al. Mapping the chemical chromatin reactivation landscape identifies BRD4-TAF1 cross-talk. *Nat Chem Biol*. 2016;12(7):504-510. doi:10.1038/nchembio.2080

18. Sekerdag E, Solaroglu I, Gursoy-Ozdemir Y. Cell Death Mechanisms in Stroke and Novel Molecular and Cellular Treatment Options. *Curr Neuropharmacol*. 2018;16(9):1396-1415. doi:10.2174/1570159X16666180302115544
